# AKT1 phosphorylates PRMT7 to promote GLUD1 methylation and gastric cancer progression

**DOI:** 10.1038/s41419-026-08601-8

**Published:** 2026-03-24

**Authors:** Ziyi Cui, Hongchen Li, Xiaoben Liang, Xinyu Zhao, Kai Xu, Yao Lu, Yan Zhang, Xia Li, Siyao Wu, Zhen Wang, Lei Lv, Yanping Xu

**Affiliations:** 1 Tongji Hospital, Frontier Science Center for Stem Cell Research, Shanghai Key Laboratory of Signalling and Disease Research, School of Life Sciences and Technology, Tongji University, Shanghai, China; 2 School of Medicine, Hebei University of Engineering, Handan, China; 3 MOE Key Laboratory of Metabolism and Molecular Medicine, Department of Biochemistry and Molecular Biology, School of Basic Medical Sciences, Fudan University, Shanghai, China

**Keywords:** Cancer metabolism, Post-translational modifications

## Abstract

Glutamine metabolism has emerged as an essential metabolic driver of tumor progression. Glutamate dehydrogenase 1 (GLUD1), a key enzyme in glutaminolysis, is frequently overexpressed in malignancies. Post-translational modifications (PTMs) are crucial for regulating protein function and tumor progression. However, the PTMs of GLUD1, particularly arginine methylation, remain unexplored. Here we report that protein arginine methyltransferase 7 (PRMT7) mediates monomethylation of GLUD1 at arginine 76 (R76), enhancing its protein stability by antagonizing ubiquitin-dependent degradation. Moreover, high glucose destabilizes GLUD1 via the PI3K/Akt pathway. Mechanistically, AKT1 phosphorylates PRMT7 at threonine 73 (T73) and promotes its activity to stabilize GLUD1 by increasing its methylation and reducing ubiquitination. Clinical analysis reveals that elevated GLUD1, PRMT7, and meGLUD1(R76) levels correlate with tumor progression in gastric cancer. In xenograft models, PRMT7 inhibitor SGC3027 combined with chemotherapeutic drugs docetaxel (DTX) synergistically suppresses tumor growth. Collectively, this study identifies the AKT1-PRMT7-GLUD1 axis as a key regulatory pathway in gastric cancer, and highlights its potential as a therapeutic target.

## Introduction

Metabolic reprogramming is a hallmark of cancer, enabling tumor cells to survive and sustain rapid proliferation. While the Warburg effect has long been recognized as the primary metabolic feature of cancer, enhanced glutamate metabolism has recently gained increasing attention for its regulatory role in cancer proliferation and metastasis. Glutamine can serve as a nitrogen source to support anabolic processes that fuel proliferation in many types of malignancies, which is also known as glutamine addiction. Several cancer cell lines, including those derived from pancreatic cancer, glioblastoma multiforme and acute myelogenous leukemia, have also demonstrated sensitivity to glutamine starvation [1]. After entering the cells, glutamine undergoes a deamination reaction catalyzed by glutaminase (GLS) to produce glutamate. Glutamate dehydrogenase 1 (GLUD1, also known as GDH1) is a key enzyme in the glutaminolysis converting glutamate to α-KG. Previous studies have demonstrated that GLUD1 provides a metabolic advantage to cancer cells through regulating carbon and nitrogen fluxes [2]. R162, a selective GLUD1 inhibitor, represses cancer cell growth and exhibits a more significant anti-cancer effect when combined with chemotherapy drugs. For instance, R162 induces anoikis in non-small-cell lung cancer (NSCLC) cells, thereby attenuating tumor metastasis. Combination of R162 with chemotherapeutic drug docetaxel (DTX) significantly inhibits tumor growth and prolongs survival of nude mice [3]. Furthermore, the activity of GLUD1 is closely associated with abnormal glucose metabolism. Current studies have shown that inhibiting glycolysis or Akt reduces GLUD1 activity [4]. Interestingly, GLUD1 promotes glucose uptake and tumor cell survival by upregulating GLUT1 under low-glucose conditions [5]. Though solid tumors frequently encounter nutrient limitations, such as glucose deprivation, recent evidence suggests that glutamine metabolism suppresses glucose uptake even when glucose availability is not a limiting factor in the tumor microenvironment [6]. However, the molecular interplay between glucose and glutamate metabolism remains poorly understood. In this study, we focus on the relationship between glucose metabolism and GLUD1. As a key signaling cascade in insulin-stimulated glycolysis, the PI3K/Akt pathway plays a pivotal role in maintaining glucose homeostasis by enhancing cellular glucose uptake and utilization. High glucose levels induce insulin resistance by downregulating Akt signaling [7], while suppressing insulin feedback enhances the efficacy of PI3K inhibitors [8]. However, whether glucose metabolism and the PI3K/Akt pathway affect GLUD1 stability and its underlining mechanism is unknown.

Post-translational modifications (PTMs) are crucial regulators of GLUD1 stability and activity. Localized in both mitochondria and cytoplasm, GLUD1 undergoes RNF213-mediated ubiquitination and translocation from mitochondria to the cytoplasm during amino acid deprivation [9]. Another E3 ligase, STUB1, promotes K48-linked ubiquitination of GLUD1, targeting it for proteasomal degradation [10]. Additionally, SIRT3-mediated deacetylation at K48 site enhances GLUD1 enzymatic activity, supporting lung adenocarcinoma survival [11]. Protein arginine methylation, mediated by protein arginine methyltransferases (PRMTs), involves the transfer of a methyl group to the guanidino nitrogen atoms of arginine residues. This modification has recently emerged as a promising therapeutic target. Overexpression of PRMTs, leading to aberrant methylation patterns, is often associated with poor prognosis in cancers [12]. While PRMTs are well known for their role in histone methylation, their regulation in non-histone protein methylation is increasingly recognized for its impact on tumor progression [13]. PRMT7, which is prominently overexpressed in breast cancer, induces epithelial-to-mesenchymal transition (EMT) and promotes metastasis by inducing E-cadherin transcription [14]. Moreover, PRMT7 facilitates mitochondrial ribosomal protein S23 (MRPS23) methylation leading to increased breast cancer cell invasion and metastasis [15]. However, the role of arginine methylation in regulating GLUD1 stability remains unexplored. Here, we report that PRMT7 catalyzes arginine methylation of GLUD1--a previously unrecognized modification. We elucidate the functional interplay between this methylation and ubiquitination, showing how they coordinately control GLUD1 protein stability.

In this study, we demonstrate that AKT1-mediated phosphorylation activates PRMT7, which stabilizes GLUD1 through methylation and suppression of ubiquitination, thereby promoting gastric cancer proliferation and metastasis. Combined treatment with the PRMT7 inhibitor SGC3027 and docetaxel (DTX) markedly inhibited tumor growth in a xenograft model, highlighting a potential therapeutic strategy for gastric cancer.

## Materials and methods

### Antibodies

Antibodies against GLUD1 (Proteintech, #14299-1-AP), PRMT7 (ABclonal, #A17164), AKT1 (Cell Signaling Technology, #4691), SYVN1 (Epizyme Biotech, # R015002), STUB1 (Epizyme Biotech, #R015148), Vinculin (Proteintech, #26520-1-AP), Mono-methyl arginine (Cell Signaling Technology, #8015), Pan phospho-Tyr (ABclonal, #AP1316), Flag-HRP (Proteintech, #HRP-66008), GFP (ABclonal, #AE078), HA-HRP (Proteintech, #HRP-81290), Ubiquitin-HRP (Cell Signaling Technology, #14049) were purchased commercially. Anti-Flag affinity gel (Beyotime, #P2282) and Anti-HA affinity gel (Beyotime, #P2287) were also purchased. To generate a site-specific antibody to detect the R76-methylated GLUD1 [meGLUD1(R76)], synthesized R76me1 peptide (GL Biochem (Shanghai) Ltd.) was coupled to KLH as an antigen to immunize two rabbits. Anti-serum was collected after five doses of immunization. The sequence of synthesized peptides was as follows:

R76me1 peptide: GFFD(R-Me)GASIVEDKLVED-Cys;

Unmodified peptide: GFFDRGASIVEDKLVED-Cys.

### Reagents

Adenosine dialdehyde (AdOx) (Sigma, #A7154), SGC3027 (MedChemExpress, #HY-112445), Compound C (MedChemExpress, #HY-13418A), Wortmannin (Topscience, #T6283), Ipatasertib dihydrochloride (Topscience, #T15374), metformin (Sangon Biotech, #A414520), insulin (Topscience, #T8221), glucose (Topscience, #T0887), docetaxel (MCE, #HY-B0011), PreScission Protease with GST tag (Beyotime Biotechnology, #P2302), Ni-NTA 6FF (His-Tag) (Sangon Biotech, #C600033) and glutathione-Sepharose 4B beads (Beyotime Biotechnology, #P2258) were commercially obtained.

### Plasmids

Full-length human GLUD1, AKT1, and PRMT1–7 cDNAs were subcloned into C-terminal Flag- or HA-tagged mammalian expression vectors (pLV-FLAG and pcDNA3.1-HA). The plasmid encoding HA-PI3KCA was purchased commercially (Miaoling Biology, #P54388). GFP-PRMT7 was kindly provided by Dr. Qunying Lei from Fudan University. The full-length coding sequences of human GLUD1 and AKT1 genes were subcloned into the pGEX-6p-1 prokaryotic expression vector, respectively. Meanwhile, the full-length coding sequence of human PRMT7 gene was subcloned into the pET28a prokaryotic expression vector. Site-directed mutagenesis was performed using KOD Plus Neo DNA polymerase (Toyobo, KOD-401) to generate: GLUD1 arginine-to-lysine mutants (R76K and R497K) and PRMT7 threonine-to-alanine mutants (T73A and T397A). All expression constructs were verified by bidirectional Sanger sequencing (Beijing Tsingke Biotech).

### Cell culture and transfection

HEK 293T (ATCC, #CRL-11268) and AGS (ATCC, #CRL-1739) cells were cultured in Dulbecco’s modified Eagle’s medium (DMEM) supplemented with 10% fetal bovine serum (FBS, Gibco, USA) in the presence of 1% penicillin and streptomycin and incubated at 37 °C in a 5% CO_2_ atmosphere while MFC cells cultured in RPMI 1640 medium correspondingly. All cell lines used in this study were confirmed to be free of mycoplasma contamination via PCR assay. For transfection with GLUD1, PRMT7, AKT1 and PI3KCA plasmid or siRNA against PRMT7, the cultured HEK 293T cells were transfected using EZ Trans Cell Transfection Reagent (Life iLab) while AGS or MFC cells were transfected using Lipofectamine^TM^ 2000 (Invitrogen), in Opti-MEM following the manufacturer’s instructions. The shRNA sequence against GLUD1 and siRNA sequences against PRMT7 were as follows:

sh*GLUD1*#1: 5′- GCCATTGAGAAAGTCTTCAAA-3′;

sh*GLUD1*#2: 5′-CCCAAGAACTATACTGATAAT-3′;

si*PRMT7*#1: 5′-UCUGUCUUUGUCAUGUAGC-3′;

si*PRMT7*#2:5′-UCAGCUAUGUUGUGGAGUU-3′;

si*PRMT7*#3: 5′-CUCGGUUUGGAGAGAUCAA-3′.

### Immunoprecipitation

Cells were lysed in ice-cold 0.5% NP-40 lysis buffer (50 mM Tris–HCl, pH 8.0, 150 mM NaCl, 0.5% NP-40, 1 mM DTT) with protease inhibitors cocktail (Selleck, #B14001; 1:100 dilution) for 40 min. After centrifugation at 12,000 rpm for 10 min at 4 °C, the supernatants were collected. For Flag- or HA-tagged protein immunoprecipitation, supernatants were incubated with anti-Flag or anti-HA beads overnight at 4 °C with gentle rotation. For endogenous immunoprecipitation, the supernatants were incubated with primary antibody at 4 °C overnight followed by incubating with Protein-A beads (Millipore, #16-663) for another 4 h at 4 °C. Immunoprecipitates were washed three times with lysis buffer, resuspended in 1× SDS loading buffer, subjected to Western blot analysis.

### RT-qPCR

Total RNA from cells were isolated using an RNA purification kit (#B0004D, EZBioscience, MN, USA) according to manufacturer’s instruction. Real-time quantitative PCRs were performed using SYBR Green (YEASEN, #11200ES03) after cDNA synthesis. Results were normalized as described in the figure legends using the ΔΔct method. ACTIN was used as an internal control. The qPCR primers used in this study are as follows:

GLUD1-forward: 5′-TTGGTAATGTGGGCCTACACTC-3′;

GLUD1-reverse: 5′-AGTCTTCCAGTTCCTTTGGGTC-3′;

ACTIN-forward: 5′-CATGTACGTTGCTATCCAGGC-3′;

ACTIN-reverse: 5′-CTCCTTAATGTCACGCACGAT-3′.

### Dot blot and antigen competition assay

R76me1 and unmodified peptides were constructed to evaluate the site specificity of meGLUD1(R76) antibodies. For dot blot assay, GLUD1 R76me1 and unmodified peptides were spotted onto a nitrocellulose membrane and air-dried for 30 min at room temperature. The membrane was then blocked with 5% non-fat milk for 1 h. For antigen competition assay, Flag-GLUD1 was immunoprecipitated and transferred to a nitrocellulose membrane. After blocking with 5% non-fat milk, each sample was blotted separately with meGLUD1(R76) antibody preincubated with either R76me1 or unmodified peptides, and the samples were processed in parallel under identical conditions.

### Recombinant protein expression and pull-down assay

Protein Expression and Purification: Recombinant vectors were transformed into E. coli BL21 (DE3) cells. Cultures were grown in LB medium (supplemented with 100 μg/mL ampicillin for pGEX-6p-1-GLUD1 and pGEX-6p-1-AKT1 or 50 μg/mL kanamycin for pET28a-PRMT7) at 37 °C until OD₆₀₀ reached 0.6–0.8. Protein expression was induced with 0.25 mM IPTG at 16 °C for 18 h. Bacteria were harvested (6000 × *g*, 10 min, 4 °C) and lysed in buffer (50 mM Tris-HCl pH 7.4, 150 mM NaCl, 1% Triton X-100, 1 mM PMSF, 1× protease inhibitor cocktail) by sonication. Soluble proteins were collected after centrifugation (12,000 × *g*, 30 min, 4 °C). Glutathione-Sepharose 4B beads (GE Healthcare) were equilibrated with lysis buffer. 50 μL of equilibrated beads were incubated with 1 mg GST-GLUD1 and GST-AKT1 lysate at 4 °C for 2 h with rotation to immobilize fusion proteins. The beads were washed five times with wash buffer (50 mM Tris-HCl pH 7.4, 150 mM NaCl, 0.1% Triton X-100) to remove non-specifically bound proteins. GST fusion proteins were eluted with 50 μL elution buffer (50 mM Tris-HCl pH 8.0, 10 mM reduced glutathione) at room temperature for 15 min. GST-tagged Precision protease (10 U, Thermo Fisher Scientific) was diluted in 100 μL of digestion buffer (50 mM Tris-HCl pH 7.0, 150 mM NaCl, 1 mM EDTA, 1 mM DTT). The mixture was incubated at 4 °C for 16 h with gentle rotation to cleave of the GST tag from the fusion proteins.

For the purification of 6×His-tagged PRMT7, the soluble cell lysate was applied to the Ni-NTA agarose resin and incubated at 4 °C for 4 h with rotation, followed by elution with 250 mM imidazole buffer. The eluate was collected for subsequent experiments.

Pull-Down Assay: Purified GLUD1 and AKT1 fusion proteins separately incubated with 6×His-tagged PRMT7 protein under gentle shaking at 4 °C for 16 h. then the reaction system was loaded onto a Ni-NTA agarose column, and the collected elution fractions were then analyzed via western blotting.

### In vitro methylation assay

HEK 293T cells overexpressing wild type Flag-GLUD1 or methyl-deficient R76K mutant or HA-PRMT7 were lysed by 0.5% NP-40 lysis buffer at 4 °C for 40 min. After centrifugation at 12,000 rpm for 10 min at 4 °C, the supernatants were incubated with Flag and HA beads at 4 °C overnight. Immunopurified proteins were mixed with methylation reaction buffer (50 mM Tris-HCl, pH 8.0, 20 mM KCl, 5 mM DTT, 4 mM EDTA) in the presence of 200 μM S-adenosyl-L-methionine (SAM, Sigma) at 37 °C for 1 h. Reactions were stopped by adding 5 × SDS-PAGE loading buffer and subjected to Western blotting analysis.

### Establishment of DTX-rapidly resistant gastric cancer

Human gastric cancer AGS cells were cultured in RPMI 1640 medium supplemented with 10% FBS. To establish a DTX‑resistant subline, a multi‑phase stepwise selection protocol was applied over an 8‑week period. Initially, cells were continuously treated with DTX at 1× IC₅₀ until reaching 70–80% confluence. The concentration was then progressively increased in subsequent phases, interspersed with intermittent high‑dose pulses and recovery periods in drug‑free medium, to allow for the adaptation and expansion of resistant populations. During the selection process, the culture medium containing DTX was replaced every 2 days, and non‑viable cells were routinely removed by gentle washing with phosphate‑buffered saline. By the final phase, resistant cells were maintained stably in medium containing DTX.

### Metformin intervention in HFD-fed mice and hepatocyte isolation

The procedures related to animal experiments were approved by Ethics Committee of Department of Laboratory Animals, Tongji University. Four-week-old C57BL/6J male mice (Charles River Labs) were acclimated for 2 weeks in the specific-pathogen-free (SPF) facility (22 ± 2 °C, 50–60% relative humidity, 12-h light/dark cycle) and fed standard chow. At the age of 6 weeks, mice were switched from standard chow to high-fat diet (HFD, 45% calories from fat) for 3 weeks. Then mice were randomly divided into two groups (*n* = 3): HFD + ddH_2_O and HFD+metformin (300 mg/kg/day via oral gavage for another 10 days). After euthanasia, primary hepatocytes were isolated via a two-step liver perfusion with HBSS and digestion buffer, followed by mechanical dissociation. The cell suspension was filtered (70 μm mesh), centrifuged (50 × *g* for 2 min), and washed twice using PBS. Then hepatocytes were lysed in RIPA lysis buffer containing protease inhibitors. The lysate was centrifuged at 12,000 × *g* for 15 min at 4 °C and the supernatant was collected. Protein concentration was determined by BCA assay. Samples were mixed with 5 × loading buffer and denatured at 100 °C for 10 min, ready for western blot analysis.

### In vitro kinase assay

Recombinant PRMT7 and AKT1 proteins were incubated together in kinase buffer (50 mM Tris-HCl, pH 7.5, 10 mM MgCl₂, 1 mM DTT) in the presence or absence of 1 mM ATP at 30 °C for 30 min. Reactions were terminated by addition of SDS sample buffer and subjected to Western blot analysis followed by immunoblotting.

### GLUD1 enzyme activity assay

AGS cells overexpressing wild type Flag-GLUD1 or methyl-deficient R76K mutant were lysed with 0.5% NP-40 lysis buffer at 4 °C for 40 min with gentle agitation to extract total cellular proteins. After centrifugation at 12,000 rpm for 10 min at 4 °C, the supernatants were incubated with Flag beads at 4°C overnight. For the measurement of GLUD1 enzymatic activity, 10 μL of the immunopurified Flag-GLUD1 proteins or R76K mutant were added to the pre-prepared reaction buffer provided in the Glutamic Acid Dehydrogenase Assay Kit (JINING Bio, Catalog No. JN24218). The reaction mixture was incubated at 37 °C for 5 min to initiate the enzyme-catalyzed reaction, and the absorbance change at the wavelength of 340 nm was recorded immediately using a microplate reader, following the detailed protocol provided with the assay kit.

### Immunohistochemistry (IHC) staining

A tissue microarray (TMA) containing 30 paired gastric carcinoma and adjacent normal tissues was constructed using specimens from patients with gastric carcinoma. Informed consent was obtained from all patients and approvals were obtained from the Ethics Committee of Tongji Hospital, Tongji University for the use of these specimens in research. Tumor tissues were fixed in 10% neutral buffered formalin for 48 h, followed by standard dehydration, paraffin embedding, and sectioning into 4-μm-thick slices. Immunohistochemical staining was performed using a streptavidin-biotin-peroxidase protocol with primary antibodies against GLUD1 (Abclonal, #A5176, 1:200), meGLUD1(R76) (personalized constructed, 1:100), and PRMT7 (Abclonal, #A17164, 1:200). Protein expression was quantified based on cytoplasmic staining intensity and the percentage of positive cells. Staining intensity was graded as: 0 (negative), 1 (weak), 2 (moderate), or 3 (strong). The percentage of positive cells was scored as: 0 (0%), 1 (1–25%), 2 (26–50%), 3 (51–75%), or 4 (76–100%). The final IHC score (range: 0–12) was calculated by multiplying intensity and percentage scores. Statistical analysis of correlations between GLUD1, PRMT7, and meGLUD1 expression levels was performed using Pearson correlation coefficients in GraphPad Prism, with statistical significance set at *p* < 0.05.

### Xenograft tumor model

The BALB/c nude mice were obtained from Shanghai Jihui Laboratory Animal Care Co. Ltd. The procedures related to animal experiments were approved by Ethics Committee of Department of Laboratory Animals, Tongji University. Following arrival, athymic nude mice were acclimated for a 2-week period in a SPF facility under controlled conditions (22 ± 2 °C, 50–60% relative humidity) with a standardized 12-h light-dark cycle.

MFC cells (2 × 10⁶ cells/mouse) were subcutaneously injected into the left groin of 5-week-old mice. When tumors reached approximately 100 mm³, mice were randomly divided into four groups (*n* = 6 per group). Experimental groups received intraperitoneal injections of 50 mg/kg SGC3027 and/or 8 mg/kg DTX at indicated time, while controls received saline treatment. Tumor volume was monitored every 2 days using caliper measurements (Volume = 0.5 × length × width²). After 12 days of treatment, the mice were euthanized, and the tumors were dissected for measurement of weight and size. Subsequently, xenograft tumors were fixed in 10% neutral buffered formalin for 48 h and then sent to ServiceBio (Wuhan, China) for Ki-67 IHC analysis (Abcam, #ab16667, 1:200 dilution).

### Cell proliferation assay

Cell proliferation was assessed using EdU incorporation and Cell Counting Kit 8 (CCK8) assays. For the EdU assay, AGS cells were seeded into 6-well plates, treated as indicated, and incubated with 50 μM EdU (YEASEN, YF594 Click-iT EdU Imaging Kit) for 2 h. Cells were then fixed with 4% formaldehyde for 15 min, permeabilized with 0.5% Triton X-100 for 20 min. After washing with PBS, Hoechst 33342 was used to stain DNA for another 30 min before imaging. EdU fluorescence intensity (EdU proportion %) was quantified using ImageJ. For the CCK8 assay, 1000 AGS cells were seeded in 96-well plates. After incubation for the indicated times, CCK8 solution (YEASEN, 40203ES76) was added to terminate the growth, and absorbance was measured at 450 nm after 2 h of incubation at 37 °C.

### Cell migration assay

For the wound healing assay, AGS cells were seeded into 6-well plates and grown to full confluence. A linear scratch was made across the monolayer using a sterile 10 μL sterile pipette tip. After gently washing twice with PBS to remove detached cells, the culture medium was replaced with serum-free DMEM. Cells were incubated for 24 h, and wound closure was monitored by imaging under a microscope. The wound area was quantified using ImageJ by measuring the distance between the wound edges at indicated time points.

### Statistical analysis

Statistical significance between experimental groups was assessed using a two-tailed unpaired Student’s *t*-test with variance homogeneity confirmed by *F*-test. Data normality was assessed using Shapiro-Wilk test prior to parametric testing. Data are presented as means ± SD from triplicate experiments. *P*-values < 0.05 were considered statistically significant (*P < 0.05, **P < 0.01, ***P < 0.001, ****P < 0.0001, n.s. = no significance). Data analysis was performed using GraphPad Prism or Image J.

## Results

### GLUD1 is methylated at arginine 76

**Fig. 1:**
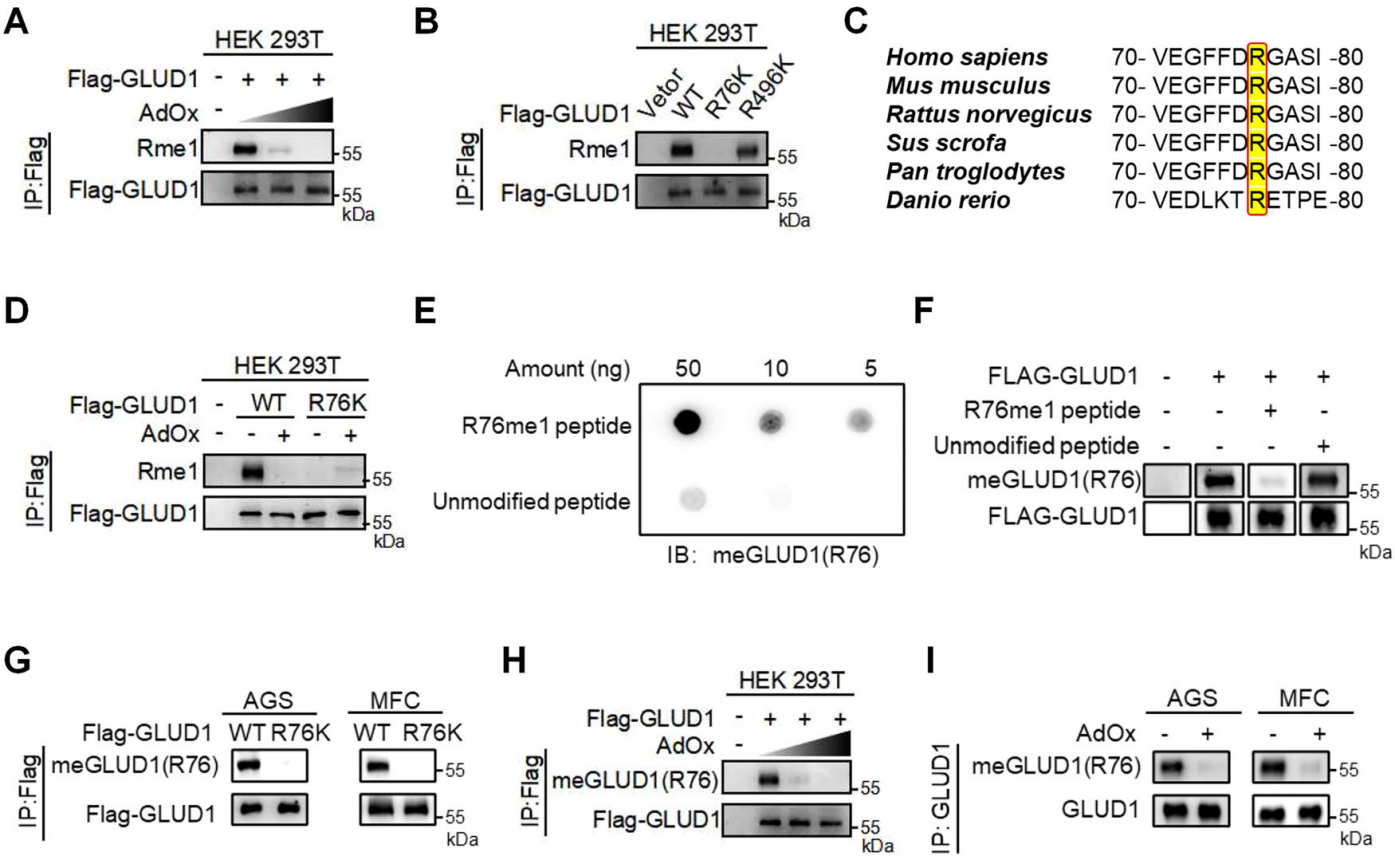
GLUD1 is methylated at arginine 76. **A** HEK 293T cells expressing Flag-tagged GLUD1 were treated with 20 μM or 50 μM AdOx for 24 h. Western blot analysis of GLUD1 methylation using a mono-methylarginine (Rme1) antibody, following immunoprecipitation with Flag beads. **B** HEK 293T cells were transfected with wild-type GLUD1 or methylation-deficient mutants (R76K, R496K) GLUD1. Western blot analysis of GLUD1 methylation after immunoprecipitation with Flag beads. **C** GLUD1 R76 is evolutionarily conserved. Amino acid sequences alignment of R76 across six mammalian species. **D** HEK 293T cells expressing either GLUD1 WT or R76K mutant were treated with or without 20 μM AdOx for 24 h. Western blot analysis of GLUD1 methylation following immunoprecipitation with Flag beads. **E** A site-specific methylation antibody (meGLUD1[R76]) specifically recognized the R76 mono-methylated (R76me1) peptide but not the unmodified peptide with dot blot assays. **F** The R76me1 peptide blocks the meGLUD1(R76) antibody but not the unmodified peptide. After incubation with unmodified peptide or R76 mono-methylated peptide (R76me1), meGLUD1(R76) antibody was used to detect immunopurified GLUD1. **G** AGS and MFC cells were transfected with wild-type GLUD1 or the R76K mutant. Western blot analysis of GLUD1 R76 methylation after immunopurified by Flag beads. **H** HEK 293T cells expressing Flag-tagged GLUD1 were treated with increasing concentrations of AdOx (0, 20 μM, 50 μM) for 24 h. Western blot analysis of R76 methylation after immunoprecipitation with Flag beads. **I** Both AGS and MFC cells were treated with 20 μM AdOx for 24 h. Western blot analysis of immunoprecipitated endogenous GLUD1 methylation.

Protein arginine methylation has recently emerged as a promising therapeutic target. To explore whether arginine methylation plays a role in glutamine metabolism, we first used Adenosine dialdehyde (AdOx), a pan PRMT inhibitor, to investigate GLUD1 methylation. Methylation of GLUD1 decreased in a dose-dependent manner after AdOx treatment of HEK 293T cells (Fig. 1A), confirming that GLUD1 is indeed methylated. To identify potential methylation sites, we queried several methylation site prediction databases, including PhosphoSitePlus, qPTM and MetOSite. The intersection of predictions identified arginine 76 (R76) and arginine 496 (R496) as potential methylation sites. To validate these sites, we mutated arginine (R) residues to lysine (K) as the methyl-deficient mutant and observed that only the R76K mutant showed a significant reduction in arginine methylation compared to wild-type GLUD1 (Fig. 1B). R76 is also a highly conserved residue, indicating the potential important role of this methylation event in various mammalian species (Fig. 1C). Furthermore, AdOx treatment significantly reduced methylation of wild-type GLUD1 but not the R76K mutant (Fig. 1D). These results indicate that GLUD1 is methylated at R76.

To specifically examine R76 methylation of GLUD1, we generated a polyclonal antibody that specifically recognizes methylated R76 [meGLUD1(R76)]. This antibody showed high specificity in both dot blot assay and antigen peptide competition assays (Fig. 1E, F). R76 methylation antibody recognized mono-methylated peptides, but not unmodified peptide, suggesting that this antibody reflects R76-monomethylated GLUD1 proteins. R76 site-specific methylation antibody efficiently recognized wild-type GLUD1, but not R76K mutant (Fig. 1G). In addition, R76 methylation levels of ectopically expressed GLUD1 was reduced under AdOx treatment (Fig. 1H). Similarly, the R76 methylation of endogenous GLUD1 decreased significantly following AdOx treatment in both AGS and MFC cells (Fig. 1I). Together, these results confirm that R76 is a major methylation site of GLUD1.

### Methylation of GLUD1 enhances protein stability

**Fig. 2:**
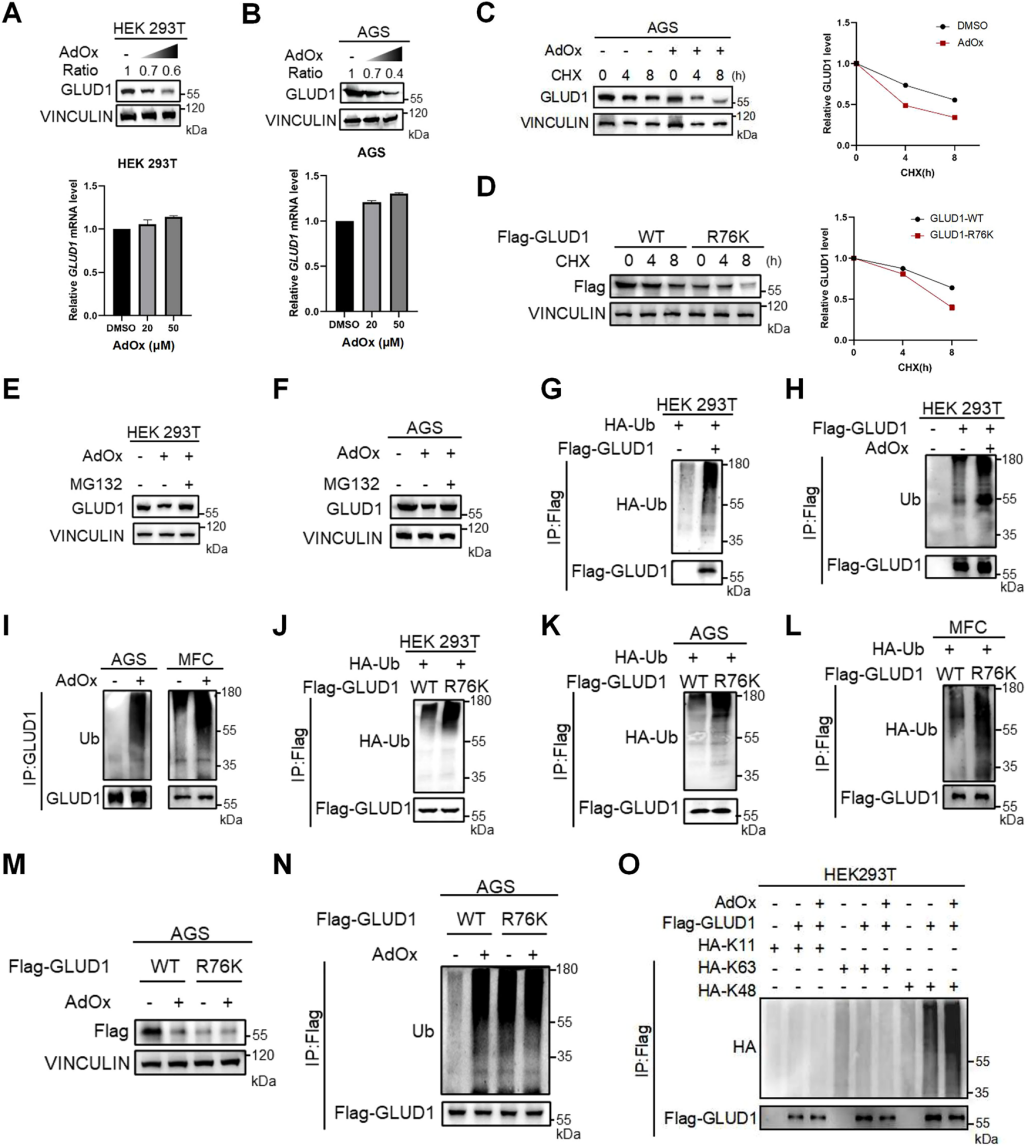
Methylation of GLUD1 enhances protein stability. HEK 293T (**A**) and AGS (**B**) cells were treated with increasing concentrations of AdOx (0, 20, 50 μM). Western blot analysis of GLUD1 protein levels and qPCR analysis of mRNA levels. The ratio represents the grayscale intensity of GLUD1 normalized to VINCULIN as measured by ImageJ. **C** AGS cells were treated with or without 20 μM AdOx for 24 h, followed by 200 μg/mL CHX treatment for the indicated time. Western blot analysis of GLUD1 protein levels. **D** AGS cells expressing wild-type GLUD1 or the R76K mutant were treated with 200 μg/mL CHX for the indicated time. Western blot analysis of GLUD1 protein levels. HEK 293T (**E**) and AGS (**F**) cells were treated with 50 μM AdOx followed by 10 μM MG132 for 4 h. Western blot analysis of GLUD1 protein levels. **G** Western blot analysis of GLUD1 ubiquitination following immunoprecipitation with Flag beads in HEK 293T cells overexpressing Flag-GLUD1 with HA-Ub. **H** HEK 293T cells transfected with Flag-GLUD1 were treated with 50 μM AdOx treatment for 24 h and 10 μM MG132 for another 4 h, followed by immunoprecipitation with Flag beads and Western blot analysis for ubiquitination. **I** AGS and MFC cells were treated similarly as (H) followed by Western blot analysis for immunopurified endogenous GLUD1 ubiquitination. Western blot analysis of GLUD1 ubiquitination after immunoprecipitation with Flag beads in HEK293T (**J**), AGS (**K**) or MFC (**L**) cells overexpressing GLUD1 WT or R76K mutant with HA-Ub. **M**, **N** AGS cells expressing wild-type GLUD1 or R76K mutant were treated with or without 50 μM AdOx for 24 h. Western blot analysis of GLUD1 protein level (**M**) and ubiquitination (**N**) after immunoprecipitation with Flag beads. **O** Western blot analysis showing ubiquitination of immunoprecipitated Flag-GLUD1 in HEK293T cells. Cells were co-transfected with HA-tagged ubiquitin mutants (K11, K63, K48) and treated with or without AdOx.

As arginine methylation plays a crucial role in regulating protein function, we first examined the effect of GLUD1 arginine methylation on protein stability. Under the treatment of AdOx with increasing concentration, GLUD1 exhibited gradual reduction in the protein level but showed no significant changes in the mRNA level (Fig. 2A, B). We also employed protein synthesis inhibitor cycloheximide (CHX) to assess the stability of GLUD1. Results showed that the half-life of GLUD1 was shortened under AdOx treatment (Fig. 2C). Similarly, the methyl-deficient R76K mutant also exhibited increased destabilization of GLUD1 compared to the wild-type GLUD1 (Fig. 2D). These results suggest that the inhibition of GLUD1 methylation decreases its protein stability.

Previous study has found that the degradation of GLUD1 is mainly dependent on ubiquitin-proteasome system [9]. To this end, we employed the proteasome inhibitor MG132 after AdOx treatment to testify GLUD1 degradation. The treatment of MG132 rescued GLUD1 protein level under AdOx treatment in both HEK 293T and AGS cells (Fig. 2E, F). After co-transfecting HA-tagged ubiquitin with Flag-tagged GLUD1, we observed that GLUD1 underwent ubiquitination modification (Fig. 2G). Treatment with AdOx increased the ubiquitination of both ectopically expressed and endogenous GLUD1, demonstrating that the inhibition of GLUD1 methylation facilitated the ubiquitin-dependent degradation of GLUD1 (Fig. 2H, I). However, the phosphorylation and acetylation level of GLUD1 protein did not exhibit significant changes under AdOx treatment (Fig. S1A). Similarly, GLUD1 R76K mutant showed higher level of ubiquitination than wild type GLUD1 (Fig. 2J–L). Furthermore, we found that AdOx treatment reduced the protein level of wild-type GLUD1, but had no effect on the methylation-deficient R76K mutant (Fig. 2M). In parallel, the ubiquitination levels of wild-type GLUD1 increased upon AdOx treatment while no significant changes were observed in the R76K mutant (Fig. 2N). Notably, GLUD1 was ubiquitinated predominantly by K48-linkage and AdOx treatment markedly enhanced K48-linked ubiquitination of GLUD1 (Fig. 2O), suggesting that methylation suppresses proteasome-targeting ubiquitin modifications. Previous studies have identified synoviolin (SYVN1) and STIP1 homology and U-box-containing protein 1 (STUB1) as key E3 ubiquitin ligases responsible for the ubiquitin-mediated proteasomal degradation of GLUD1 [10, 16]. To further dissect the molecular mechanism underlying GLUD1 degradation via the proteasomal pathway, we sought to identify the mediator(s) that govern the ubiquitin-dependent degradation of GLUD1 under conditions where GLUD1 methylation is abrogated. SYVN1 displayed the increased binding to GLUD1 following AdOx treatment, suggesting that SYVN1 may mediate the AdOx-induced GLUD1 ubiquitination (Fig. S1B). Collectively, these findings indicate that GLUD1 arginine methylation enhances protein stability by restraining the ubiquitin-proteasome mediated degradation.

### PRMT7 directly methylates GLUD1 at R76

**Fig. 3:**
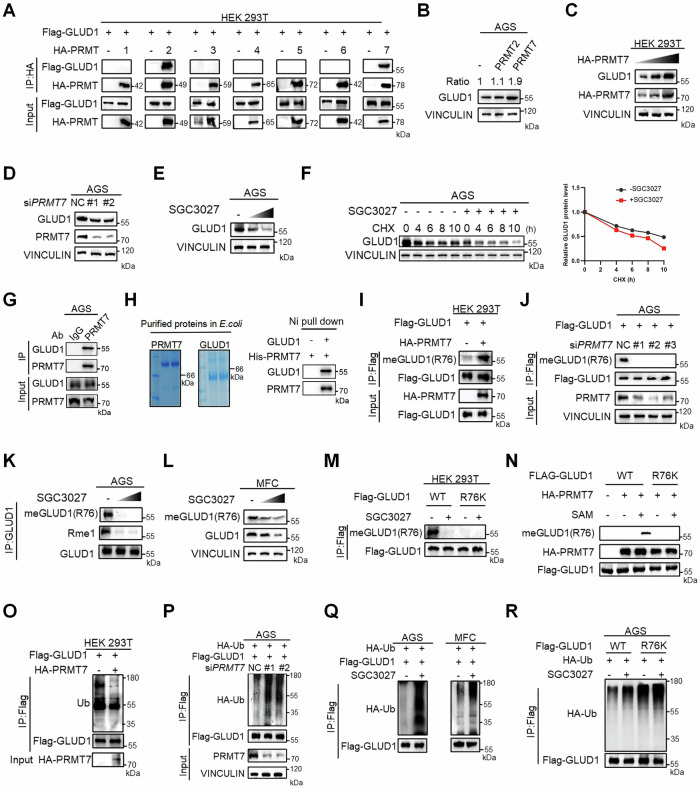
PRMT7 directly methylates GLUD1 at R76. **A** Western blot analysis of interactions following co-immunoprecipitation between Flag-GLUD1 and HA-PRMT1-7 with Flag beads. **B** Western blot analysis of GLUD1 levels in AGS cells with overexpression of HA-PRMT2 or HA-PRMT7. The ratio represents the grayscale intensity of GLUD1 normalized to VINCULIN as measured by ImageJ. **C** Western blot analysis of GLUD1 levels in HEK293T cells expressing 1, 2 or 5 μg HA-PRMT7. **D** Western blot analysis of GLUD1 levels in AGS cells transfected with control or PRMT7 siRNAs. **E** Western blot analysis of GLUD1 levels from AGS cells treated with SGC3027 (0, 5, 10 μM) for 24 h. **F** Western blot and quantitative analysis of GLUD1 protein levels in the presence or absence of SGC3027 by 200 μg/mL CHX treatment for the indicated time. **G** Western blot analysis of endogenous interaction between PRMT7 and GLUD1 in AGS cells. **H** Pull down assay to analyze the interaction between GLUD1 and PRMT7. Recombinant GLUD1 and PRMT7 purified from *E. coli* were subjected to binding assays. **I** Western blot analysis of R76 methylation of immunopurified Flag-GLUD1 from AGS cells co-expressing HA-PRMT7. **J** Western blot analysis of R76 methylation in AGS cells transfected with PRMT7 siRNAs. **K** Western blot analysis of R76 methylation of immunopurified GLUD1 from AGS cells treated with SGC3027 (0, 5, 10 μM) for 24 h. **L** Western blot analysis of endogenous GLUD1 R76 methylation in MFC cells treated similarly as (**K**). **M** Western blot analysis of R76 methylation of immunopurified Flag-GLUD1 WT or R76K mutant from HEK293T cells treated with or without 10 μM SGC3027 for 24 h. **N** Immunopurified Flag-GLUD1 WT or R76K mutant with HA-PRMT7 were incubated with SAM, followed by western blot analysis of R76 methylation. **O** Western blot analysis of immunopurified Flag-GLUD1 ubiquitination from HEK293T cells co-expressing with or without HA-PRMT7. **P** Western blot analysis of immunopurified Flag-GLUD1 ubiquitination from AGS cells co-expressing with HA-Ub and PRMT7 siRNAs. **Q** AGS and MFC cells co-expressing Flag-GLUD1 and HA-Ub were treated with or without 10 μM SGC3027 for 24 h followed by western blot analysis of immunopurified Flag-GLUD1 ubiquitination. **R** AGS cells co-expressing HA-Ub and either GLUD1 WT or R76K mutant were treated similarly as (Q). Western blot analysis of immunopurified Flag-GLUD1 ubiquitination.

As GLUD1 methylation could enhance protein stability, we aimed to identify the methyltransferase responsible for this modification. Protein arginine methyltransferases (PRMTs) are key enzymes that catalyze arginine methylation. We first performed co-immunoprecipitation (co-IP) assays using Flag-tagged GLUD1 and HA-tagged PRMTs (PRMT1-7). Result showed that GLUD1 interacted with both PRMT2 and PRMT7(Fig. 3A); however, only PRMT7 markedly increased GLUD1 protein levels (Fig. 3B). Overexpression of PRMT7 enhanced GLUD1 protein levels in a dose-dependent manner (Fig. 3C), whereas PRMT7 knockdown led to a notable decrease in GLUD1 protein levels (Fig. 3D). Similarly, treatment with the PRMT7-specific inhibitor SGC3027 gradually reduced GLUD1 expression (Fig. 3E) and accelerated GLUD1 degradation rate (Fig. 3F). Together these findings suggest that PRMT7 interacts with GLUD1 and enhances its protein stability. To validate the interaction between GLUD1 and PRMT7, we performed endogenous immunoprecipitation, as well as pull down assay in vitro using recombinant proteins purified from *E. coli*. Both of the results consistently demonstrated the direct interaction between GLUD1 and PRMT7 (Fig. 3G, H). Next, we employed methylation-specific antibody targeting meGLUD1(R76) to detect the direct catalytic activity of PRMT7 on GLUD1. Immunoblot analysis revealed that overexpression of PRMT7 in HEK 293T cells increased GLUD1 methylation at R76 site (Fig. 3I). Conversely, both PRMT7 knockdown and SGC3027 treatment led to a reduction in GLUD1 R76 methylation levels (Fig. 3J–L). Consistently, treatment with SGC3027 remarkably reduced wild-type GLUD1 methylation at R76 site, but not its R76K mutant (Fig. 3M). We also conducted in vitro methylation assay to detect the immunopurified GLUD1 methylation level. Wild-type GLUD1 showed increase in R76 methylation in the presence of S-adenosyl-L-methionine (SAM) as the methyl donor, but not the R76K mutant (Fig. 3N). These results demonstrate that PRMT7 directly mediates GLUD1 methylation at R76 site.

As previously mentioned, GLUD1 R76 methylation enhances its protein stability by inhibiting the ubiquitin-dependent degradation. Here we aimed to explore the crosstalk between PRMT7-mediated GLUD1 methylation and ubiquitination. Results showed that GLUD1 ubiquitination level decreased significantly under the overexpression of PRMT7 (Fig. 3O). In contrast, PRMT7 inhibition through either PRMT7 knockdown (Fig. 3P) or pharmaceutical inhibition using SGC3027 (Fig. 3Q) markedly enhanced GLUD1 ubiquitination, indicating methylation-ubiquitination crosstalk. Moreover, the treatment of SGC3027 raised wild-type GLUD1 ubiquitination level but did not affect the R76K mutant (Fig. 3R). These findings confirm that PRMT7-mediated GLUD1 methylation at R76 residue improves its protein stability by supressing GLUD1 ubiquitin-dependent degradation.

### High glucose destabilizes GLUD1 via PI3K/Akt pathway

**Fig. 4:**
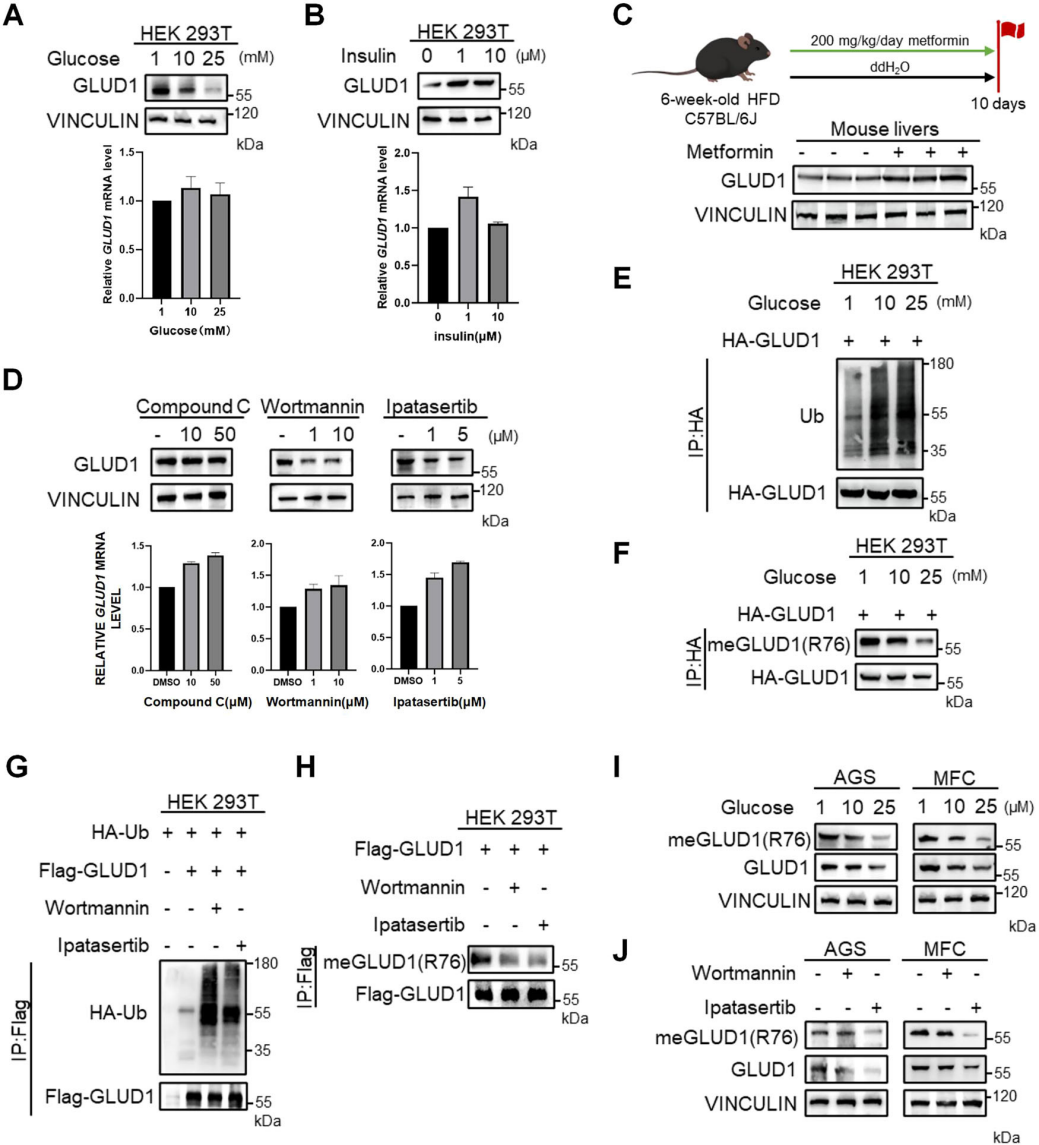
High glucose destabilizes GLUD1 via PI3K/Akt pathway. **A** Western blot of GLUD1 levels in HEK 293T cells treated with 1, 10, 25 mM glucose for 24 h and qPCR analysis of mRNA levels. **B** Western blot analysis of GLUD1 levels in HEK 293T cells with 0, 1, 10 μM insulin after overnight starvation and qPCR analysis of mRNA level. **C** Western blot analysis of GLUD1 protein levels in HFD-fed C57BL/6 J mouse livers after 10 days metformin treatment. **D** Western blot analysis of GLUD1 and qPCR analysis of mRNA levels after Compound C (AMPK inhibitor), Wortmannin (PI3K inhibitor) and Ipatasertib (AKT inhibitor) for 24 h with indicated concentration. **E**, **F** HEK 293T cells expressing HA-GLUD1 were treated with increasing glucose as indicated for 24 h. Western blot analysis of GLUD1 ubiquitination (**E**) and methylation (**F**) against meGLUD1(R76) antibodies after immunoprecipitation with HA beads. **G**, **H** HEK 293T cells expressing Flag-GLUD1 with HA-Ub were treated with or without Wortmannin or Ipatasertib for 24 h. Western blot analysis of GLUD1 ubiquitination (**G**) and R76 methylation (**H**) levels. **I** Western blot analysis of GLUD1 methylation against meGLUD1(R76) antibodies in AGS or MFC cells with increasing glucose as indicated for 24 h. **J** Western blot analysis of GLUD1 methylation against meGLUD1(R76) antibodies in AGS or MFC cells treated with or without Wortmannin or Ipatasertib for 24 h.

In addition to the well-established Warburg effect, glutamine metabolism has emerged as a critical pathway for providing energy and nutrients in cancer cells [1]. GLUD1, a key enzyme in glutaminolysis, is a promising therapeutic target. Here we sought to elucidate the interplay between glucose and glutamine metabolism in tumorigenesis, with a particular focus on the regulation of GLUD1. We designed a glucose concentration gradient ranging from 1 mM to 25 mM and observed a dose-dependent reduction in GLUD1 protein abundance with increasing glucose concentrations. Importantly, no significant changes were detected in GLUD1 mRNA level (Fig. 4A). Consistent with the glucose-deprivation phenotype, insulin treatment, a potent activator of glucose utilization, significantly increased GLUD1 protein levels without altering mRNA expression (Fig. 4B). To extend these findings in vivo, we analyzed liver tissues from high-fat diet (HFD) mice treated with metformin, a pharmacological agent that lowers systemic glucose by suppressing hepatic gluconeogenesis [17]. Results showed that metformin increased hepatic GLUD1 protein expression in HFD mice (Fig. 4C). Together, these results suggest that low glucose availability enhances GLUD1 protein stability. Previous studies have shown that high glucose downregulates both PI3K/Akt and AMPK signaling pathway [18]. To further investigate the signaling pathway involved, we employed specific inhibitors of AMPK (Compound C), PI3K (Wortmannin), and AKT (Ipatasertib). Interestingly, GLUD1 protein level was decreased significantly under Wortmannin and Ipatasertib treatment without altering mRNA level while AMPK inhibition had no significant effect (Fig. 4D). These results indicate that the PI3K/Akt pathway plays a predominant role in maintaining GLUD1 stability, consistent with the observation that high glucose decreases GLUD1 protein expression.

As previously mentioned, GLUD1 stability is regulated by methylation and ubiquitination. We analyzed the level of GLUD1 ubiquitination and methylation at different glucose concentrations and in the presence of PI3K and AKT inhibitors. High glucose levels increased GLUD1 ubiquitination while reducing methylation in a dose-dependent manner (Fig. 4E, F). Similar observations were obtained under PI3K and AKT inhibition (Fig. 4G, H). Using the site-specific antibody recognizing methylated GLUD1, we confirmed that high-glucose conditions markedly reduced endogenous GLUD1 R76 methylation (Fig. 4I). Notably, Akt inhibition caused a more pronounced reduction in both total GLUD1 protein levels and R76 methylation than PI3K inhibition, suggesting that Akt may more directly regulate GLUD1 methylation (Fig. 4J). Given that PI3K/Akt pathway is inhibited under high glucose, these results suggest that high glucose disturbs PI3K/Akt pathway thereby destabilizing GLUD1 by reducing its methylation and increasing ubiquitination.

### AKT1 enhances the stability of GLUD1 by phosphorylating PRMT7

**Fig. 5:**
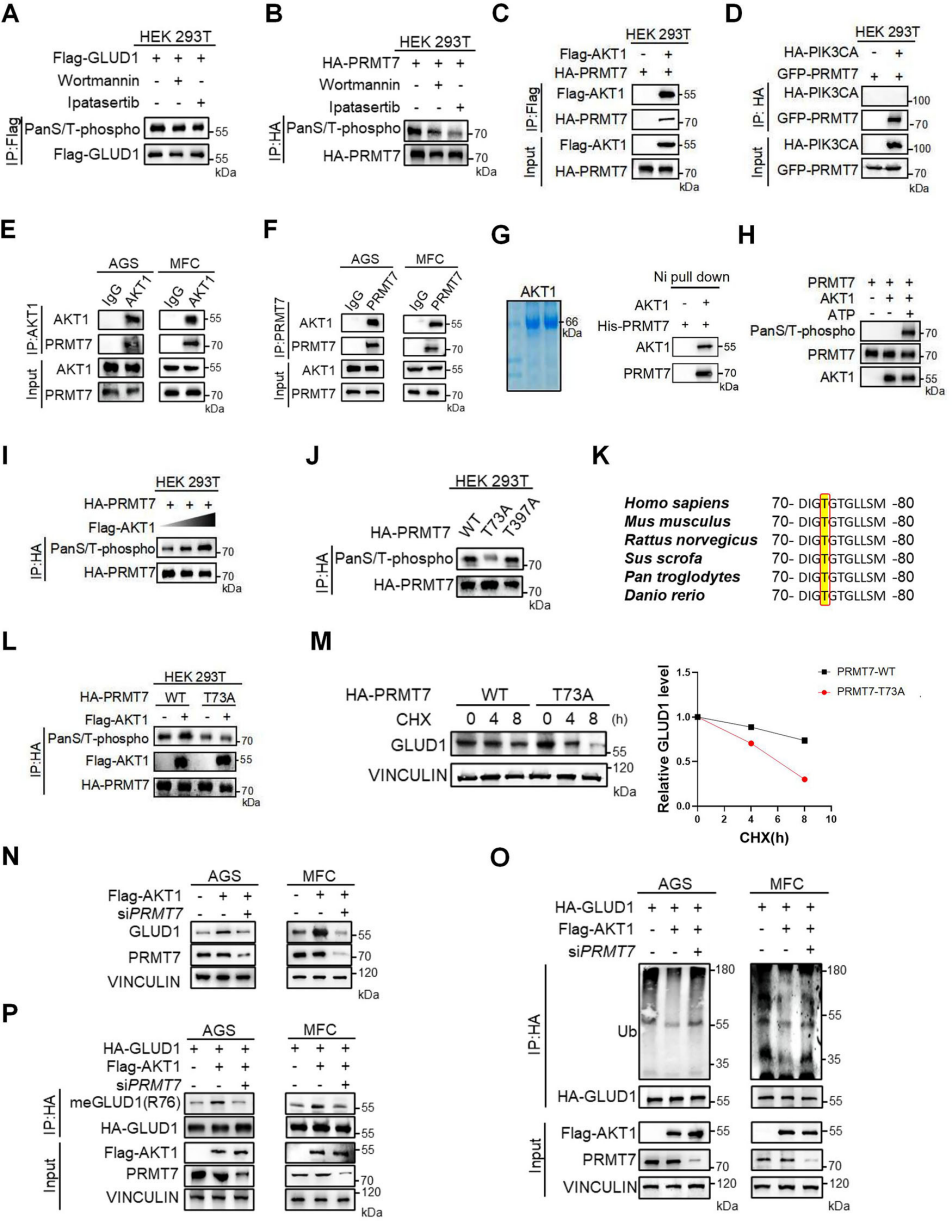
AKT1 enhances the stability of GLUD1 by phosphorylating PRMT7. **A**, **B** HEK293T cells expressing Flag-GLUD1 (A) or HA-PRMT7 (B) were treated with 10 μM Wortmannin or Ipatasertib for 24 h. Western blot analysis of phosphorylation levels following immunoprecipitation with Flag or HA beads. Western blot analysis of interactions of HA/GFP-PRMT7 with Flag-AKT1 (**C**) or HA-PIK3CA (**D**) in HEK293T cells. Endogenous AKT1 (**E**) or PRMT7 (**F**) was pulled down using specific antibodies from AGS or MFC cells. Western blot analysis of input and immunoprecipitates. **G** Pull down assay to analyze the interaction between AKT1 and PRMT7. Recombinant AKT1 and PRMT7 purified from *E. coli* were subjected to binding assays. **H** In vitro kinase assays were conducted by incubating recombinant PRMT7 and AKT1 in the presence or absence of ATP. Phosphorylation was assessed using a pan-serine/threonine phospho antibody. **I** Western blot analysis of immunopurified HA-PRMT7 phosphorylation from HEK293T cells co-transfected with increasing Flag-AKT1 (1, 2, 5 μg). **J** Western blot analysis of phosphorylation levels of immunopurified HA-PRMT7 WT, T73A and T397A mutants from HEK293T cells. **K** Evolutionary conservation of PRMT7 T73. Amino acid sequences alignment of T73 across six mammalian species. **L** Western blot analysis of phosphorylation levels of immunopurified HA-PRMT7 WT or T73A mutant from HEK 293T cells co-expressing with or without Flag-AKT1. **M** AGS cells expressing PRMT7 WT or T73A mutant were treated with 200 μg/mL cycloheximide for the indicated times. Western blot analysis of GLUD1 protein levels. **N** Flag-AKT1 with or without PRMT7 siRNA was transfected into AGS or MFC cells. Western blot analysis of GLUD1 protein levels. **O** HA-GLUD1 was co-transfected with or without Flag-AKT1 and PRMT7 siRNA into AGS or MFC cells. Western blot analysis of immunopurified HA-GLUD1 ubiquitination. **P** AGS or MFC cells were treated same as (O). Western blot analysis of immunopurified HA-GLUD1 methylation.

Next, we aimed to elucidate the molecular mechanism by which the PI3K/Akt pathway regulates GLUD1 stabilization. The PI3K/Akt pathway is aberrantly activated in gastric cancer and promotes tumorigenesis by phosphorylating downstream effectors [19]. Therefore, we examined the phosphorylation of GLUD1 and PRMT7 upon the PI3K/Akt inhibition and found that PRMT7 phosphorylation was markedly reduced, whereas GLUD1 phosphorylation remained unchanged (Fig. 5A, B). To identify the kinase responsible for PRMT7 phosphorylation within the PI3K/Akt pathway, we performed co-immunoprecipitation assays between PRMT7 and either PIK3CA or AKT1. The results demonstrated that PRMT7 specifically interacted with AKT1 but not PIK3CA (Fig. 5C, D). Endogenous co-immunoprecipitation confirmed the interaction between PRMT7 and AKT1 in both AGS and MFC cells (Fig. 5E, F). Consistently, in vitro pull-down assays using recombinant proteins demonstrated a direct association between PRMT7 and AKT1 (Fig. 5G). Meanwhile, we also conducted in vitro kinase assays and validated that AKT1 phosphorylates PRMT7 (Fig. 5H), suggesting the key role of AKT1 in regulating PRMT7 phosphorylation. Moreover, the phosphorylation of PRMT7 was elevated in an AKT1 dose-dependent manner (Fig. 5I). Subsequently, using the PhosphoSitePlus, EPSD, and dbPAF databases for phosphorylation site prediction, we identified threonine 73 (Thr73) and threonine 397 (Thr397) of PRMT7 as potential phosphorylation sites. To investigate the functional role of these residues, we generated phosphorylation-deficient mutants by site-directed mutagenesis, substituting threonine (T) with alanine (A) (designated as T73A and T397A, respectively). Notably, phosphorylation was decreased significantly in PRMT7 T73A mutant and this T73 residue was highly conserved across species (Fig. 5J, K). Structurally, T73 site is located within a flexible coil region that is likely solvent-exposed, potentially facilitating conformational recognition by AKT1. Consistently, AKT1 specifically increased the phosphorylation of wild-type PRMT7 but not the T73A mutant (Fig. 5L), suggesting that AKT1 phosphorylates PRMT7 at T73 site. Next, we ectopically overexpressed wild-type PRMT7 and T73A mutant and performed CHX assay to observe GLUD1 degradation. The half-life was prolonged when overexpressing wild-type PRMT7 compared to T73A mutant (Fig. 5M). Collectively, these findings indicate that AKT1 phosphorylates PRMT7 at threonine 73, which is critical for subsequent regulation of GLUD1 stability.

To further investigate the role of PRMT7 in AKT1-induced GLUD1 stabilization, we overexpressed AKT1 and simultaneously knocked down PRMT7 in AGS and MFC cells. While AKT1 overexpression alone enhanced GLUD1 stability, PRMT7 knockdown attenuated the stabilizing effect, leading to a decrease in GLUD1 protein levels (Fig. 5N). As previously mentioned, the stability of GLUD1 is regulated by the crosstalk of its ubiquitination and methylation modifications. Consistently, the overexpression of AKT1 alone decreased GLUD1 ubiquitination while knockdown of PRMT7 significantly increased GLUD1 ubiquitination levels (Fig. 5O). Similarly, compared to the significant increase in GLUD1 methylation caused by the overexpression of AKT1 alone, co-knockdown of PRMT7 reversed this effect, leading to a reduction in GLUD1 methylation (Fig. 5P). Taken together, these findings reveal that AKT1 directly phosphorylates PRMT7 at threonine 73 and then PRMT7 phosphorylation promotes GLUD1 stability by augmenting GLUD1 methylation while concomitantly suppressing its ubiquitination.

### PRMT7-mediated R76 methylation of GLUD1 promotes gastric cancer proliferation and migration

Given that GLUD1 catalyzes the conversion of glutamate to α-ketoglutarate (α-KG) to replenish the tricarboxylic acid (TCA) cycle, we determined whether GLUD1 methylation regulates the enzymatic activity and metabolic flux in gastric cancer cells. The results showed that methyl-deficient R76K mutant significantly decreased the enzymatic activity of GLUD1 in comparison with the wild-type (WT) (Fig. S2A). The targeted metabolomics analyses revealed that the overall glutamine metabolism, TCA cycle flux and nucleotide synthesis decreased in cells expressing GLUD1 methyl-deficient R76K mutant compared to those expressing wild-type GLUD1 (Fig. S2B, C). Collectively, these data demonstrate that GLUD1 methylation acts as a metabolic switch, enhancing glutamine-driven TCA anaplerosis and nucleotide synthesis, thereby supporting tumor growth.

**Fig. 6:**
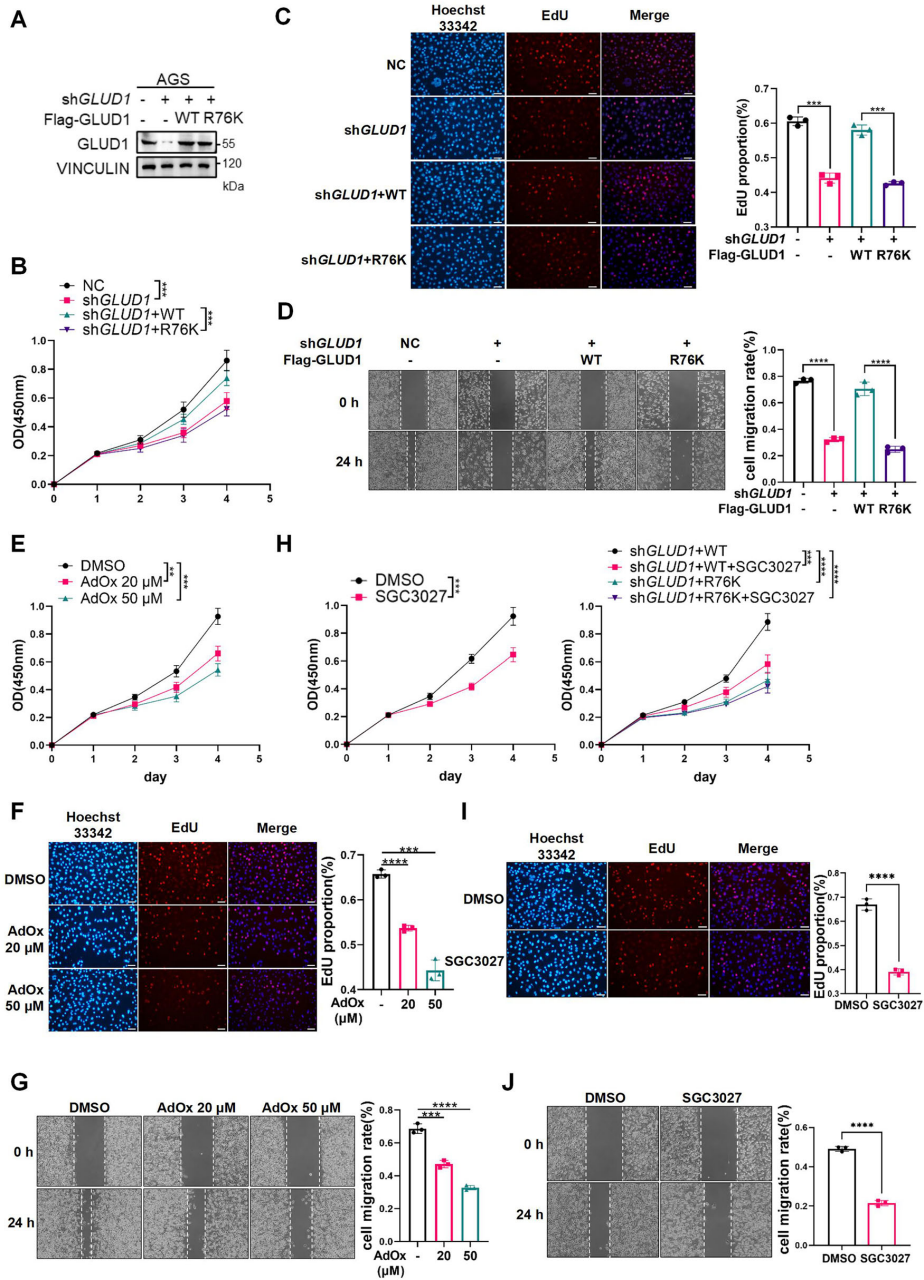
PRMT7-mediated R76 methylation of GLUD1 promotes gastric cancer proliferation and migration. **A–D** GLUD1 knockdown reduces gastric cancer cell proliferation and migration, which is rescued by re-expression of wild-type GLUD1 but not R76K mutant. Western blot analysis of Flag-GLUD1 (WT/R76K) re-expression in AGS cells following GLUD1 knockdown (**A**); Proliferation rates by CCK-8 assay (**B**) and EdU-positive cell quantification (**C**); Wound healing assay with migration rates quantified (**D**). **E**–**G** AdOx suppresses cell proliferation and migration dose-dependently. AGS cells were treated with or without 20 or 50 μM AdOx for 24 h followed by CCK8 assay (**E**), EdU assay (**F**) and wound healing assay (**G**). **H**–**J** SGC3027 suppresses proliferation and migration. AGS cells or GLUD1 knockdown cells with re-expression of GLUD1 WT or R76K mutant were treated with or without 10 μM SGC3027 for 24 h followed by CCK8 assay (**H**), EdU assay (**I**) and wound healing assay (**J**). Scale bar: 50 μm. Data are presented as mean ± SD. (*n* = 3, unpaired two-tailed *t*-test, **p < 0.01, ***p < 0.001, ****p < 0.0001).

To delineate the functional role of PRMT7-mediated GLUD1 methylation in gastric cancer progression, we conducted CCK8 and EdU assays to assess cell proliferation, along with wound healing assays to evaluate migration. Results showed that GLUD1 knockdown decreased proliferation rates in AGS cells. Re-introduction of wild-type GLUD1 rescued the suppression while the R76K mutant failed to compensate (Fig. 6A–C). Similarly, GLUD1 knockdown impaired cell migration, which was restored by wild-type GLUD1 but not by the R76K mutant (Fig. 6D). Moreover, we conducted pharmacological blockade of GLUD1 methylation using AdOx (20, 50 μM) or SGC3027 (10 μM) to evaluate cell proliferation and migration. AdOx treatment reduced cell proliferation, as shown in CCK8 and EdU assays (Fig. 6E, F), and also impaired AGS cell migration (Fig. 6G). SGC3027 also similarly suppressed proliferation and migration (Fig. 6H–J). However, its anti-proliferative activity was restricted to cells re-expressing wild-type GLUD1 but not the R76K mutant following GLUD1 knockdown.

Collectively, these findings identify PRMT7-mediated methylation of GLUD1 at R76 as a critical driver of gastric cancer cell proliferation and migration, suggesting a potential therapeutic vulnerability.

### PRMT7-mediated GLUD1 methylation enhances gastric cancer growth and targeting PRMT7 boosts the therapeutic efficacy of DTX

**Fig. 7:**
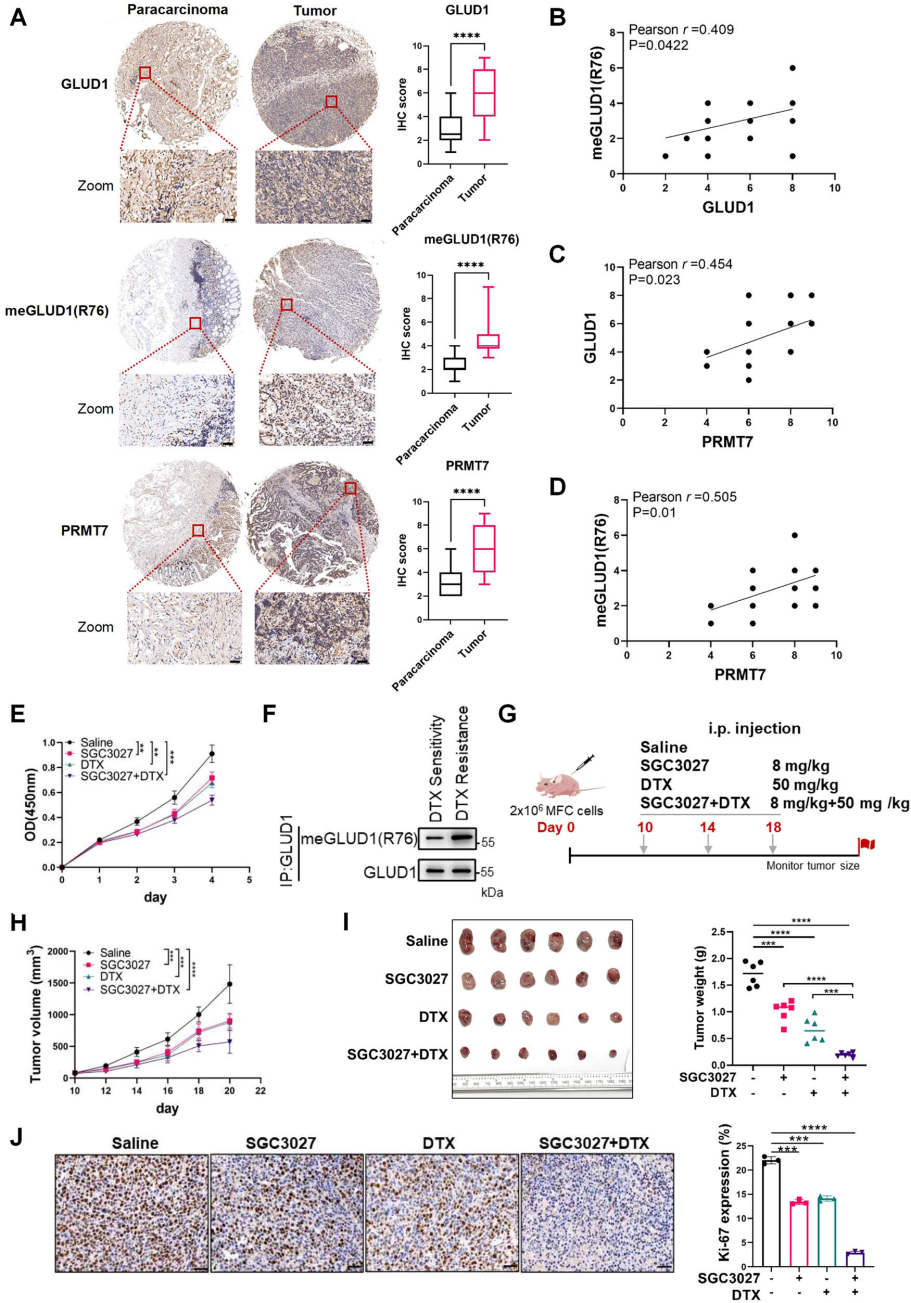
PRMT7-mediated GLUD1 methylation enhances gastric cancer growth and targeting PRMT7 boosts the therapeutic efficacy of DTX. **A** Representative immunohistochemical (IHC) staining of PRMT7, GLUD1, and meGLUD1(R76) in human gastric paracarcinoma and tumor tissues. Scale bar: 50 μm. IHC scores were calculated as the percentage of positively stained tumor cells (mean ± SD, *n* = 30, unpaired two-tailed *t*-test, ****p < 0.0001). **B**–**D** Positive correlation among GLUD1, meGLUD1(R76), and PRMT7 in gastric cancer. Pearson correlation coefficients (*r*) were calculated for 30 biologically independent samples. Overlapping data points are indicated by proportional symbol sizing. **E** CCK8 assay in AGS cells treated with SGC3027 (10 μM), DTX (100 nM) or a combination. (mean ± SD, *n* = 3, unpaired two-tailed *t*-test, **p < 0.01, ***p < 0.001, n.s. = no significance) **F** Western blot analysis of GLUD1 methylation in DTX-sensitive and DTX-resistant gastric cancer cells. **G**–**I** MFC cells (2.5 × 10⁶) were subcutaneously injected into 5-week-old nude mice (*n* = 6 per group). Mice were treated with SGC3027 (10 mg/kg), DTX (8 mg/kg), or a combination as indicated (**G**). Tumor volume was measured every 2 days (**H**) and tumor weight was quantified at endpoint (**I**). (mean ± SD, unpaired two-tailed *t*-test, ***p < 0.001, ****p < 0.0001). **J** Representative IHC images and quantification of Ki-67 staining in xenograft tumors from each treatment group. Scale bar: 50 μm. (*n* = 3, mean ± SD, unpaired two-tailed *t*-test, ***p < 0.001, ****p < 0.0001).

To explore the clinical relevance of PRMT7-mediated GLUD1 methylation, we performed immunohistochemistry (IHC) analysis on a tissue microarray (TMA) consisting of 30 paired gastric carcinoma and adjacent normal tissues samples. Tumors exhibited significantly higher expression of GLUD1, meGLUD1(R76), and PRMT7 compared to normal tissues (Fig. 7A). Pearson correlation analysis revealed positive correlations between GLUD1 and meGLUD1(R76) (*r* = 0.409, *p* = 0.0422), GLUD1 and PRMT7 (*r* = 0.454, *p* = 0.023), and meGLUD1(R76) and PRMT7 (*r* = 0.505, *p* = 0.01) (Fig. 7B–D). These results support that PRMT7-mediated GLUD1 methylation enhances its stability and promote tumor progression in human gastric cancer.

As previously mentioned, PRMT7-mediated methylation enhances GLUD1 protein stability and promotes gastric cancer cell proliferation. Given that R162, a specific inhibitor of GLUD1, has been reported to suppress tumor growth and enhance the efficacy of chemotherapeutic agents such as DTX [3]. We hypothesized that PRMT7 specific inhibitor SGC3027 would exert similar tumor-suppressive effects. As reported, SGC3027 is the first selective and cell-active PRMT7 inhibitor [20]. We therefore evaluated whether combining SGC3027 with DTX could achieve comparable or superior therapeutic effects. To this end, we first assessed the impact of SGC3027 alone or in combination with DTX on AGS cell proliferation using CCK8 assays. Results showed that SGC3027 significantly inhibited AGS cell proliferation, and the combination of SGC3027 with DTX exhibited a further enhanced inhibitory effect (Fig. 7E), suggesting a synergistic anti-tumor activity of SGC3027 and DTX. To further explore the mechanistic basis of this synergy, we examined GLUD1 methylation in DTX-resistant gastric cancer cells and found that meGLUD1(R76) levels were markedly elevated compared with DTX-sensitive cells (Fig. 7F), supporting the role of GLUD1 methylation in driving metabolic adaptation to chemotherapy. Next, we examined the therapeutic potential of SGC3027 and DTX in vivo establishing xenograft tumor models in nude mice. Mice bearing xenograft tumors were randomly divided into four treatment groups: saline, SGC3027, DTX or a combination of SGC3027 and DTX. Both SGC3027 and DTX were administered via intraperitoneal injection at indicated time points. Both SGC3027 and DTX monotherapies suppressed tumor growth compared to the control while the combination group exhibited a synergistic suppression (Fig. 7G, H). Notably, tumors from the combination group showed an approximately 80% reduction in weight compared to the saline-treated controls (Fig. 7I). Immunohistochemical analysis of Ki-67, a proliferation marker, further confirmed diminished proliferative activity in tumors from the dual treatment of SGC3027 and DTX (Fig. 7J). These findings collectively suggest that PRMT7 inhibition enhances the therapeutic efficacy of DTX, demonstrating a synergistic effect in suppressing tumor growth. Mechanistically, PI3K/Akt pathway is activated under low glucose. Activated AKT1 phosphorylates PRMT7 at T73 residue thereby increasing the GLUD1 stabilization through PRMT7-mediated methylation and inhibiting GLUD1 ubiquitin-dependent degradation, which ultimately facilitates gastric cancer cell proliferation and migration. Targeting this AKT1-PRMT7-GLUD1 axis with SGC3027 enhances the therapeutic response to DTX, providing a novel combinatorial therapy for gastric cancer.

## Discussion

In this study, we report arginine methylation at R76 as a novel PTM of GLUD1, which shields it from ubiquitin-dependent proteolysis. Using a custom-developed meGLUD1(R76) antibody, we validated this modification and identified PRMT7 as the dedicated methyltransferase targeting R76. Mechanistically, PRMT7-mediated methylation of GLUD1 inhibits its K48-linkage ubiquitination, thereby stabilizing the protein.

Emerging evidence highlights the critical role of glutamine as an alternative carbon source for bioenergetics and anabolic biosynthesis in addition to glucose [21–23]. To investigate glucose levels and GLUD1 regulation, we established glucose concentration gradients and observed that high glucose destabilized GLUD1 by inhibiting the PI3K/AKT pathway, whereas glucose downregulation, achieved by insulin or metformin treatment, promoted GLUD1 protein accumulation. Given that the PI3K/AKT pathway functions through phosphorylation cascades [24], we investigated the phosphorylation status of GLUD1 and PRMT7. Mechanistically, AKT1, not PIK3CA, directly binds to and phosphorylates PRMT7 at T73. The AKT1-PRMT7-GLUD1 axis governs the stability of GLUD1 as PRMT7 knockdown not only reduces GLUD1 methylation but also promotes its ubiquitination, leading to decreased protein levels. Of note, although PRMT7 T73 site does not conform to the canonical AKT1 consensus motif, it resides within a flexible coil region that is likely solvent-exposed, making it accessible for AKT1-mediated phosphorylation. A similar non-canonical substrate recognition mechanism has been reported for insulin receptor substrate 1 (IRS-1), where conformational flexibility facilitates phosphorylation at structurally accessible sites [25, 26].

Furthermore, our study reveals significant upregulation of GLUD1 under low-glucose conditions, indicating a novel metabolic plasticity by which tumor cells reprogram glutamine metabolism to adapt to glucose scarcity. These findings align with recent evidence that tumors preferentially uptake glutamine over glucose in resource-constrained microenvironments [27]. The enhanced GLUD1 expression suggests a selective advantage for tumors to exploit glutamine as an alternative fuel, extending the classical Warburg effect by highlighting compensatory glutaminolysis under low-glucose conditions. Notably, our metabolomic profiling revealed that PRMT7-mediated GLUD1 methylation functions as a critical node governing metabolic plasticity. Specifically, GLUD1 methylation enhanced the overall glutamine metabolism, TCA cycle and nucleotide synthesis in gastric cancer (Fig. S2B, C). This metabolic rewiring suggests that the AKT1-PRMT7-GLUD1 axis not only stabilizes GLUD1 but also orchestrates glutamine flux toward energy production and biosynthetic pathways that sustain tumor growth, providing a multi-layered mechanism underlying gastric cancer progression.

Given the frequent overexpression of GLUD1 in various malignancies [6, 28–30], targeting the down-regulation of GLUD1 is regarded as a promising therapeutic target. To examine the role GLUD1 in cancer progression, we performed cell migration and cell viability assay in vitro. The inhibition of GLUD1 methylation, either pharmaceutical inhibition using AdOx or SGC3027 or genetically via GLUD1 knockdown significantly suppressed cell proliferation in CCK8 and EdU assay as well as impaired cell migration in wound healing assays. Clinically, analysis of 30 paired gastric cancer tissues revealed elevated expression of GLUD1, meGLUD1(R76) and PRMT7 in tumor tissues compared to adjacent normal tissues. Pearson correlation analysis confirmed a positive association between GLUD1 and meGLUD1(R76), validating R76 as a critical methylation site in tumor progression. Furthermore, PRMT7 expression correlated positively with both GLUD1 and meGLUD1(R76), supporting its role in stabilizing GLUD1 via R76 methylation. R162, a GLUD1 selective inhibitor, has been reported to suppress cancer cell proliferation and enhance the efficacy of chemotherapeutic drug DTX [2, 29, 31]. Combination of R162 and DTX exhibits synergetic resistance in malignant progression in metastasis and invasion [3]. In this study, we replaced R162 with the PRMT7 inhibitor SGC3027 and found that co-treatment with SGC3027 and DTX produced comparable anti-tumor efficacy. It turned out that the combination of SGC3027 and DTX suppressed tumor growth more effectively than either treatment alone. Moreover, GLUD1 methylation was elevated in DTX-resistant cells, suggesting that GLUD1 methylation may contribute to chemoresistance and that its inhibition can enhance docetaxel efficacy.

Our work defines PRMT7-dependent GLUD1 methylation as a critical post-translational regulator of gastric cancer progression, though the precise mechanisms coordinating methylation and ubiquitination require further exploration. Strikingly, we uncovered the AKT1-PRMT7-GLUD1 axis as a critical controller of tumor growth, providing a potential target and strategy for clinical treatment of gastric cancer.

## Supplementary information

Original western blots-main figures

Original western blots-supplement

Supplementary Figure with figure legend

## Data Availability

The original western blot images and microscopy data reported in this paper is displayed in the supplementary figures. No original code was used in this study. Any additional information required to reanalyze the data reported in this paper is available from the corresponding authors upon request. More detailed materials are in the supplementary file.
