# Supplementary material for: AKT1 phosphorylates PRMT7 to promote GLUD1 methylation and gastric cancer progression
Source: Cell Death Dis. 2026 Mar 24;17(1):363. doi: 10.1038/s41419-026-08601-8 (PMC13040045; doi:10.1038/s41419-026-08601-8)
Supplement: Supplementary file 1 — Original western blots-main figures [file 41419_2026_8601_MOESM1_ESM.pdf]

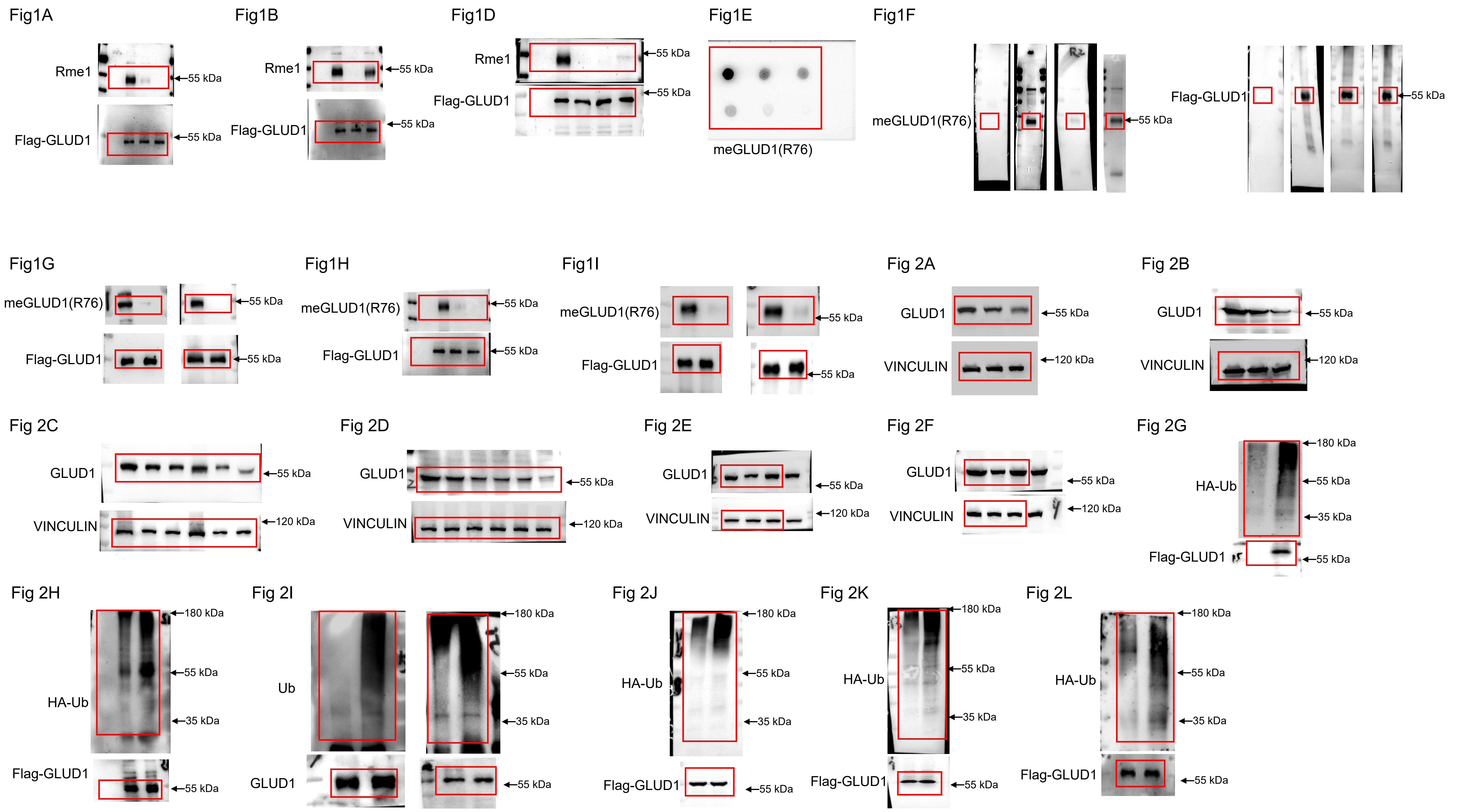
Fig1A
Rme1
←55 kDa
Flag-GLUD1
←55 kDa
Fig1B
Rme1
←55 kDa
Flag-GLUD1
←55 kDa
Fig1D
Rme1
←55 kDa
Flag-GLUD1
←55 kDa
Fig1E
meGLUD1(R76)
Fig1F
meGLUD1(R76)
←55 kDa
Flag-GLUD1
←55 kDa
Fig1G
meGLUD1(R76)
←55 kDa
Flag-GLUD1
←55 kDa
Fig1H
meGLUD1(R76)
←55 kDa
Flag-GLUD1
←55 kDa
Fig1I
meGLUD1(R76)
←55 kDa
Flag-GLUD1
←55 kDa
Fig 2A
GLUD1
←55 kDa
VINCULIN
←120 kDa
Fig 2B
GLUD1
←55 kDa
VINCULIN
←120 kDa
Fig 2C
GLUD1
←55 kDa
VINCULIN
←120 kDa
Fig 2D
GLUD1
←55 kDa
VINCULIN
←120 kDa
Fig 2E
GLUD1
←55 kDa
VINCULIN
←120 kDa
Fig 2F
GLUD1
←55 kDa
VINCULIN
←120 kDa
Fig 2G
HA-Ub
←180 kDa
←55 kDa
←35 kDa
Flag-GLUD1
←55 kDa
Fig 2H
HA-Ub
←180 kDa
←55 kDa
←35 kDa
Flag-GLUD1
←55 kDa
Fig 2I
Ub
←180 kDa
←55 kDa
←35 kDa
GLUD1
←55 kDa
Fig 2J
HA-Ub
←180 kDa
←55 kDa
←35 kDa
Flag-GLUD1
←55 kDa
Fig 2K
HA-Ub
←180 kDa
←55 kDa
←35 kDa
Flag-GLUD1
←55 kDa
Fig 2L
HA-Ub
←180 kDa
←55 kDa
←35 kDa
Flag-GLUD1
←55 kDa

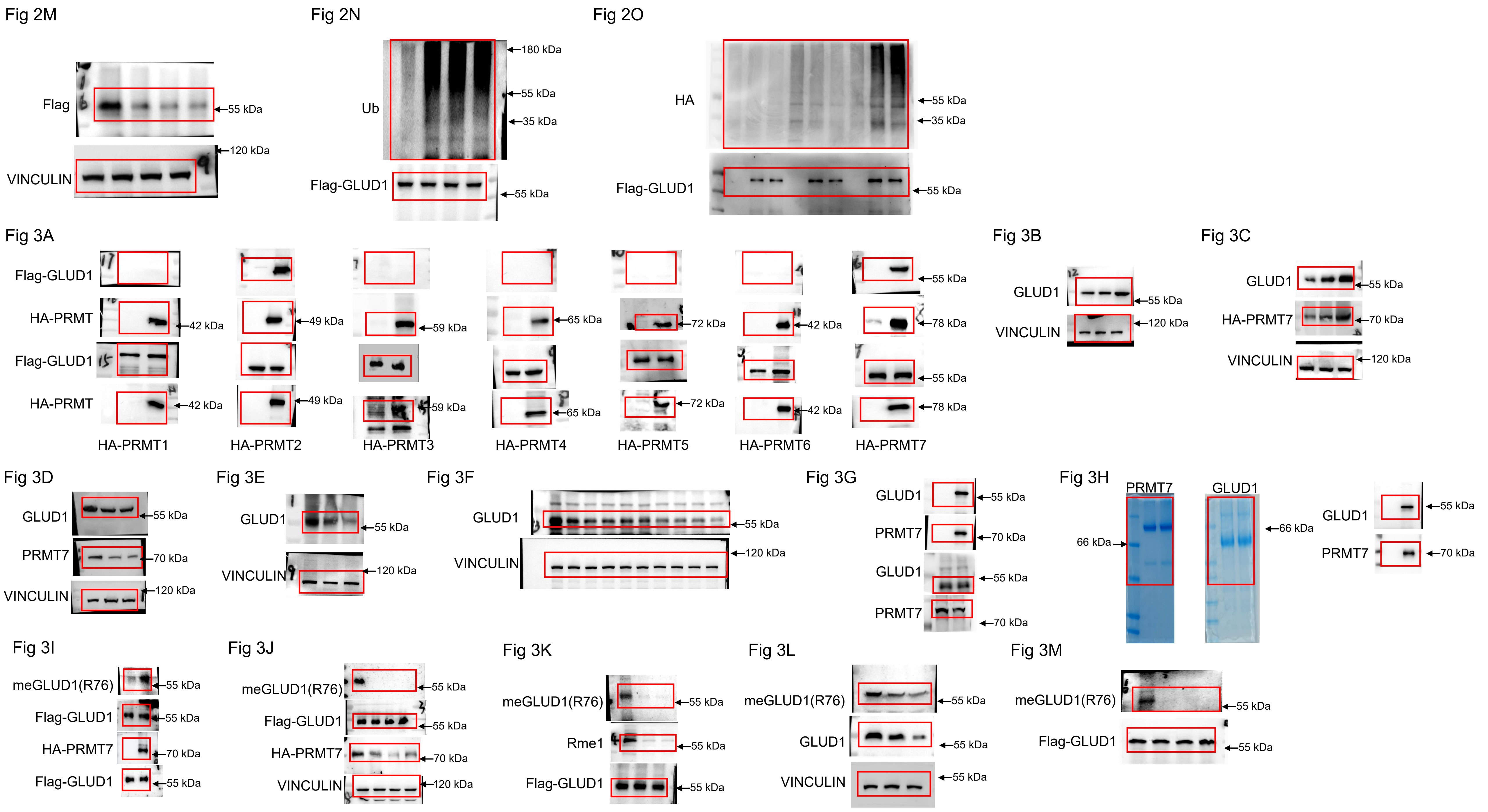

Fig 2M
Flag
55 kDa
VINCULIN
120 kDa
Fig 2N
Ub
180 kDa
55 kDa
35 kDa
Flag-GLUD1
55 kDa
Fig 2O
HA
55 kDa
35 kDa
Flag-GLUD1
55 kDa
Fig 3A
Flag-GLUD1
HA-PRMT
Flag-GLUD1
HA-PRMT
42 kDa
42 kDa
49 kDa
49 kDa
59 kDa
59 kDa
65 kDa
65 kDa
72 kDa
72 kDa
42 kDa
42 kDa
55 kDa
78 kDa
55 kDa
78 kDa
HA-PRMT1
HA-PRMT2
HA-PRMT3
HA-PRMT4
HA-PRMT5
HA-PRMT6
HA-PRMT7
Fig 3B
GLUD1
55 kDa
VINCULIN
120 kDa
Fig 3C
GLUD1
55 kDa
HA-PRMT7
70 kDa
VINCULIN
120 kDa
Fig 3D
GLUD1
55 kDa
PRMT7
70 kDa
VINCULIN
120 kDa
Fig 3E
GLUD1
55 kDa
VINCULIN
120 kDa
Fig 3F
GLUD1
55 kDa
VINCULIN
120 kDa
Fig 3G
GLUD1
55 kDa
PRMT7
70 kDa
GLUD1
55 kDa
PRMT7
70 kDa
Fig 3H
PRMT7
GLUD1
66 kDa
66 kDa
GLUD1
55 kDa
PRMT7
70 kDa
Fig 3I
meGLUD1(R76)
55 kDa
Flag-GLUD1
55 kDa
HA-PRMT7
70 kDa
Flag-GLUD1
55 kDa
Fig 3J
meGLUD1(R76)
55 kDa
Flag-GLUD1
55 kDa
HA-PRMT7
70 kDa
VINCULIN
120 kDa
Fig 3K
meGLUD1(R76)
55 kDa
Rme1
55 kDa
Flag-GLUD1
55 kDa
Fig 3L
meGLUD1(R76)
55 kDa
GLUD1
55 kDa
VINCULIN
55 kDa
Fig 3M
meGLUD1(R76)
55 kDa
Flag-GLUD1
55 kDa

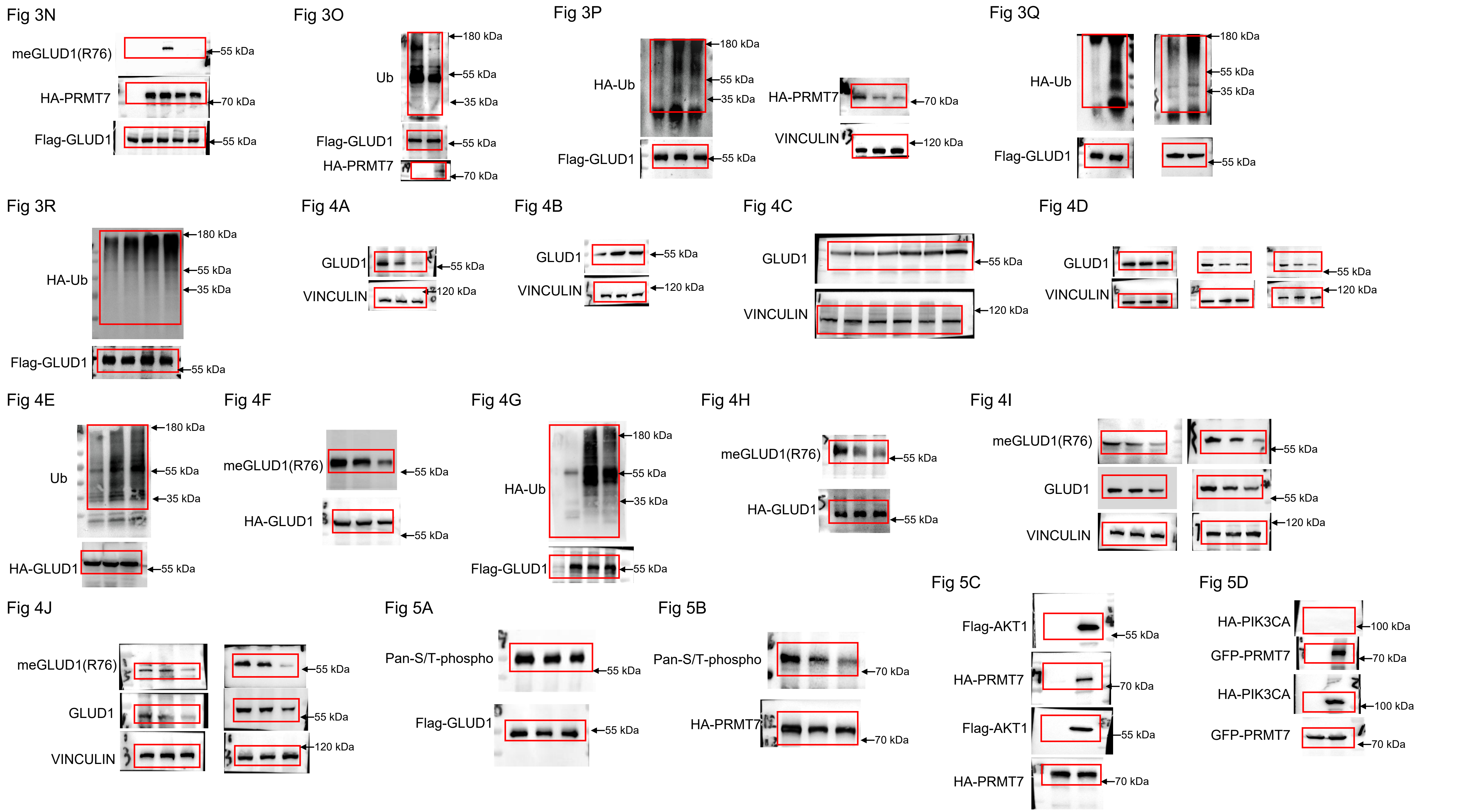

Fig 3N
meGLUD1(R76)
55 kDa
HA-PRMT7
70 kDa
Flag-GLUD1
55 kDa
Fig 3O
Ub
180 kDa
55 kDa
35 kDa
Flag-GLUD1
55 kDa
HA-PRMT7
70 kDa
Fig 3P
HA-Ub
180 kDa
55 kDa
35 kDa
Flag-GLUD1
55 kDa
HA-PRMT7
70 kDa
VINCULIN
120 kDa
Fig 3Q
HA-Ub
180 kDa
55 kDa
35 kDa
Flag-GLUD1
55 kDa
Fig 3R
HA-Ub
180 kDa
55 kDa
35 kDa
Flag-GLUD1
55 kDa
Fig 4A
GLUD1
55 kDa
VINCULIN
120 kDa
Fig 4B
GLUD1
55 kDa
VINCULIN
120 kDa
Fig 4C
GLUD1
55 kDa
VINCULIN
120 kDa
Fig 4D
GLUD1
55 kDa
VINCULIN
120 kDa
Fig 4E
Ub
180 kDa
55 kDa
35 kDa
HA-GLUD1
55 kDa
Fig 4F
meGLUD1(R76)
55 kDa
HA-GLUD1
55 kDa
Fig 4G
HA-Ub
180 kDa
55 kDa
35 kDa
Flag-GLUD1
55 kDa
Fig 4H
meGLUD1(R76)
55 kDa
HA-GLUD1
55 kDa
Fig 4I
meGLUD1(R76)
55 kDa
GLUD1
55 kDa
VINCULIN
120 kDa
Fig 4J
meGLUD1(R76)
55 kDa
GLUD1
55 kDa
VINCULIN
120 kDa
Fig 5A
Pan-S/T-phospho
55 kDa
Flag-GLUD1
55 kDa
Fig 5B
Pan-S/T-phospho
70 kDa
HA-PRMT7
70 kDa
Fig 5C
Flag-AKT1
55 kDa
HA-PRMT7
70 kDa
Flag-AKT1
55 kDa
HA-PRMT7
70 kDa
Fig 5D
HA-PIK3CA
100 kDa
GFP-PRMT7
70 kDa
HA-PIK3CA
100 kDa
GFP-PRMT7
70 kDa

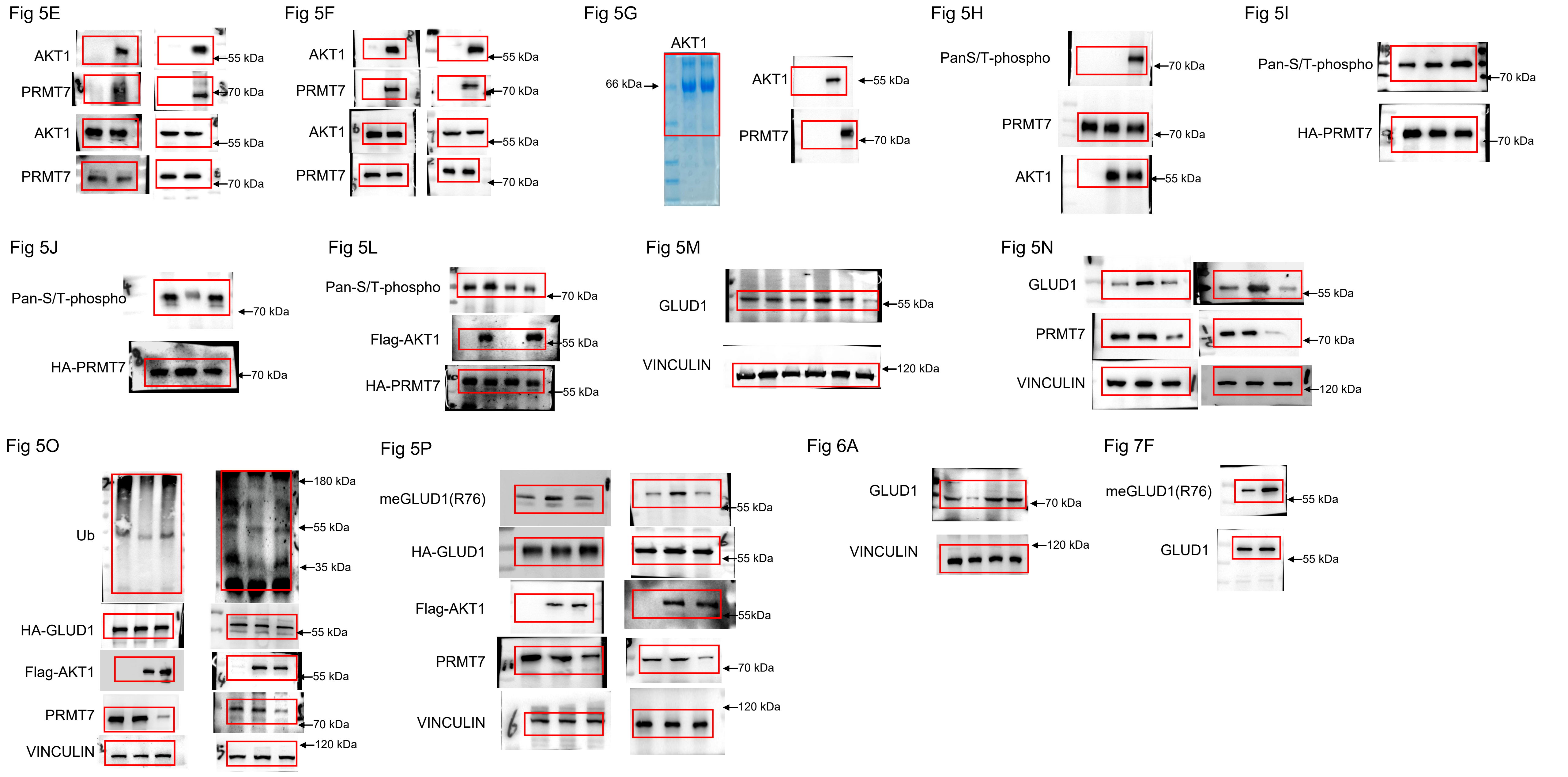
Fig 5E
AKT1
PRMT7
AKT1
PRMT7
55 kDa
70 kDa
55 kDa
70 kDa
Fig 5F
AKT1
PRMT7
AKT1
PRMT7
55 kDa
70 kDa
55 kDa
70 kDa
Fig 5G
AKT1
66 kDa
AKT1
PRMT7
55 kDa
70 kDa
Fig 5H
PanS/T-phospho
PRMT7
AKT1
70 kDa
70 kDa
55 kDa
Fig 5I
Pan-S/T-phospho
HA-PRMT7
70 kDa
70 kDa
Fig 5J
Pan-S/T-phospho
HA-PRMT7
70 kDa
70 kDa
Fig 5L
Pan-S/T-phospho
Flag-AKT1
HA-PRMT7
70 kDa
55 kDa
55 kDa
Fig 5M
GLUD1
VINCULIN
55 kDa
120 kDa
Fig 5N
GLUD1
PRMT7
VINCULIN
55 kDa
70 kDa
120 kDa
Fig 5O
Ub
HA-GLUD1
Flag-AKT1
PRMT7
VINCULIN
180 kDa
55 kDa
35 kDa
55 kDa
55 kDa
70 kDa
120 kDa
Fig 5P
meGLUD1(R76)
HA-GLUD1
Flag-AKT1
PRMT7
VINCULIN
55 kDa
55 kDa
55kDa
70 kDa
120 kDa
Fig 6A
GLUD1
VINCULIN
70 kDa
120 kDa
Fig 7F
meGLUD1(R76)
GLUD1
55 kDa
55 kDa
