# Supplementary figures and images for: AKT1 phosphorylates PRMT7 to promote GLUD1 methylation and gastric cancer progression
Source: Cell Death Dis. 2026 Mar 24;17(1):363. doi: 10.1038/s41419-026-08601-8 (PMC13040045; doi:10.1038/s41419-026-08601-8)

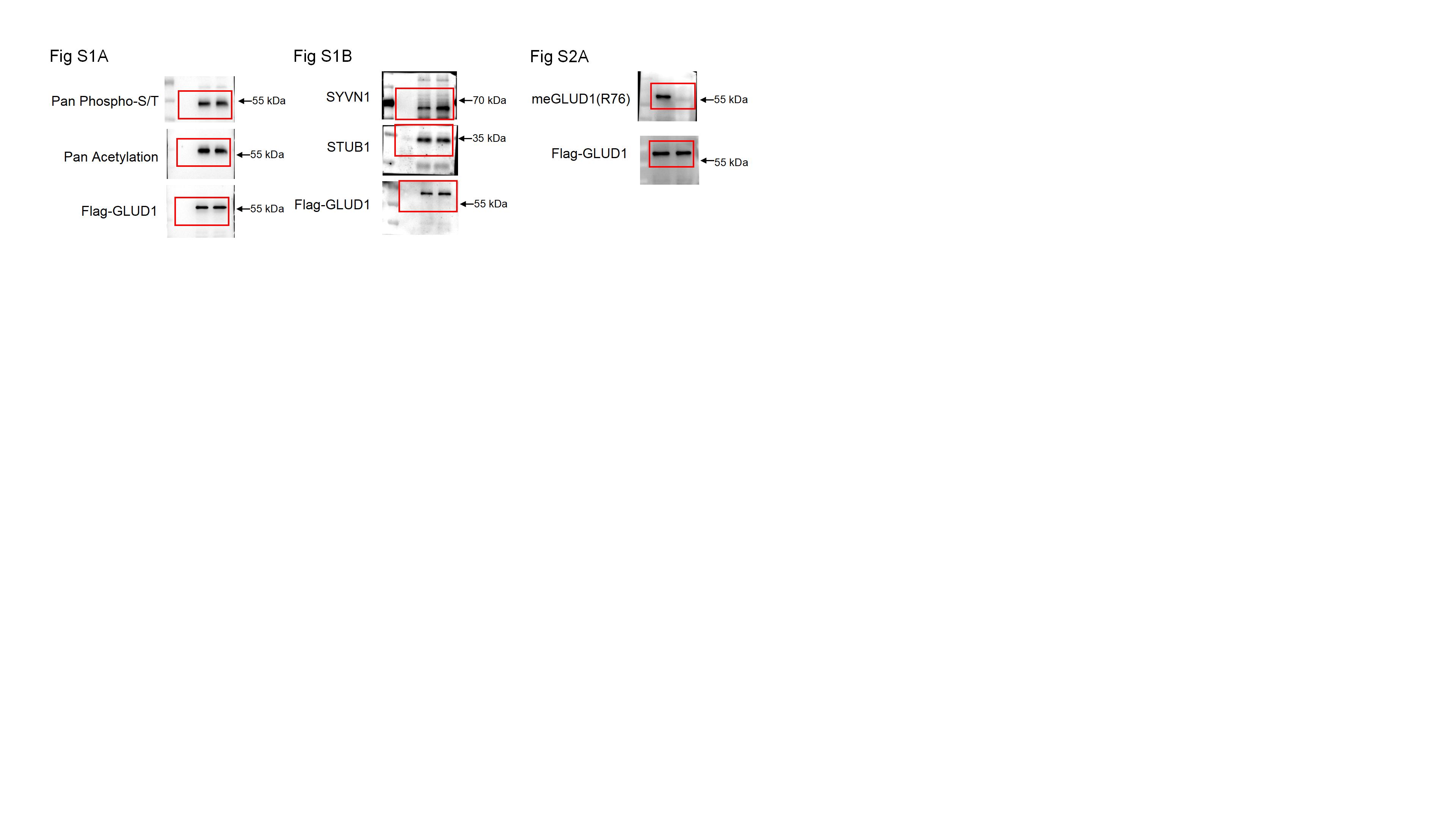

Supplement: Supplementary file 2 — Original western blots-supplement [file 41419_2026_8601_MOESM2_ESM.jpg]
