# Supplementary material for: AKT1 phosphorylates PRMT7 to promote GLUD1 methylation and gastric cancer progression
Source: Cell Death Dis. 2026 Mar 24;17(1):363. doi: 10.1038/s41419-026-08601-8 (PMC13040045; doi:10.1038/s41419-026-08601-8)
Supplement: Supplementary file 3 — Supplementary Figure with figure legend [file 41419_2026_8601_MOESM3_ESM.docx]

**Supplementary Figures**

**
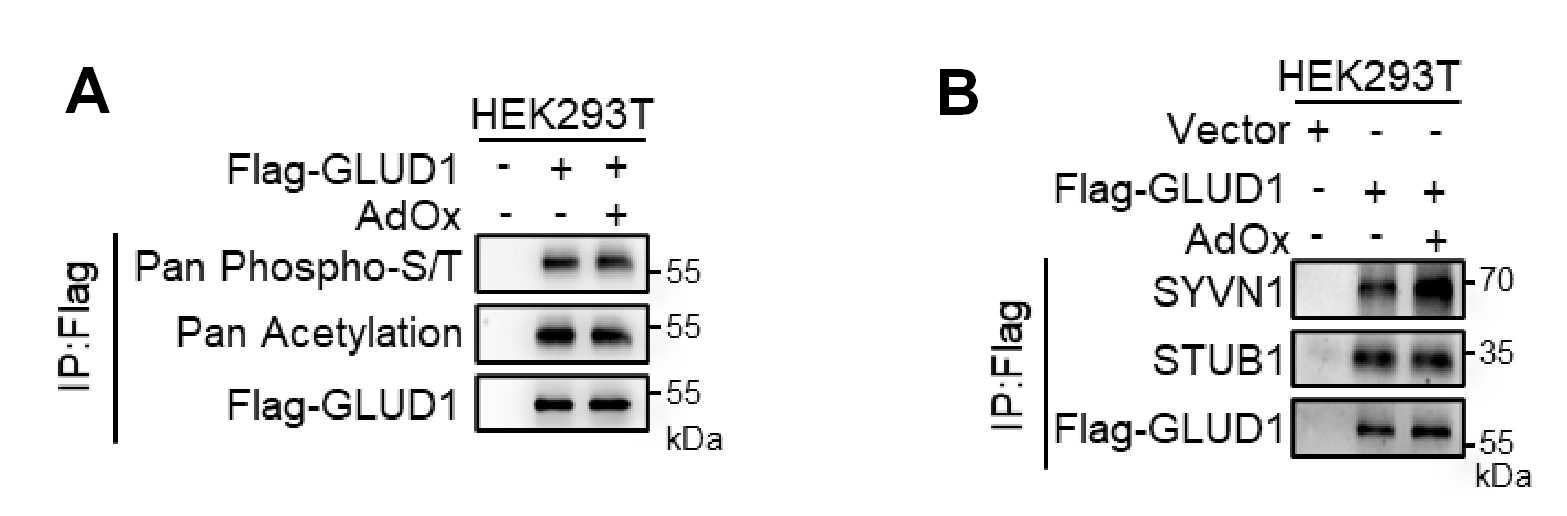
**

**Fig. S1: Effect of AdOx on GLUD1 post-translational modifications (phosphorylation and acetylation) and potential E3 ligase binding.**

(A) Western blot analysis of immunoprecipitated Flag-GLUD1 phosphorylation and acetylation under AdOx treatment. (B) Western blot analysis of interaction between GLUD1 with potential E3 ligases (SYVN1 and STUB1) after immunoprecipitation with Flag beads.

**Fig. S2: Methylation of GLUD1 affects glutamine metabolism, TCA cycle and nucleotide synthesis in gastric cancer cells.**

(A) Enzymatic activity analysis of wild-type GLUD1 and R76A mutant (upper panel). Western blot analysis of R76 methylation of immunopurified Flag-GLUD1 and R76A mutant (lower panel). (B) Schematic representation of metabolic pathways from glutamine into the tricarboxylic acid (TCA) cycle and purine and pyrimidine synthesis. OAA, oxaloacetate; AKG, α-ketoglutarate; CAD, Carbamoyl-phosphatesynthetase2, Aspartate transcarbamylase, and Dihydroorotase; GOT1, Glutamic-Oxaloacetic Transaminase 1; GLS, Glutaminase; PPAT, Phosphoribosyl Pyrophosphate Amidotransferase. (C) Bar graph depicting the relative levels of metabolites in glutamine metabolism, TCA cycle and nucleotide synthesis. Significance was defined as P < 0.05 and fold change (FC) > 1.5.
